# A Novel Algicidal Bacterium, *Microbulbifer* sp. YX04, Triggered Oxidative Damage and Autophagic Cell Death in *Phaeocystis globosa*, Which Causes Harmful Algal Blooms

**DOI:** 10.1128/spectrum.00934-21

**Published:** 2022-01-12

**Authors:** Xiaoying Zhu, Shuangshuang Chen, Guiying Luo, Wei Zheng, Yun Tian, Xueqian Lei, Luming Yao, Caiming Wu, Hong Xu

**Affiliations:** a State Key Laboratory of Cellular Stress Biology, School of Life Sciences, Xiamen University, Xiamen, China; b School of Life Sciences, Xinjiang Normal University, Urumqi, China; c Laboratory of Marine Environmental Science and Key Laboratory of the Ministry of Education for Coastal and Wetland Ecosystems, Xiamen, China; University of Michigan-Ann Arbor

**Keywords:** *Microbulbifer* sp. YX04, *Phaeocystis globosa*, algicidal bacterium, autophagic cell death, harmful algal bloom, transcriptome analysis

## Abstract

Phaeocystis globosa causes severe marine pollution by forming harmful algal blooms and releasing hemolytic toxins and is therefore harmful to marine ecosystems and aquaculture industries. In this study, *Microbulbifer* sp. YX04 exerted high algicidal activity against P. globosa by producing and secreting metabolites. The algicidal activity of the YX04 supernatant was stable after exposure to different temperatures (−80 to 100°C) and pH values (4 to 12) for 2 h, suggesting that algicidal substances could temporarily be stored under these temperature and pH value conditions. To explore the algicidal process and mechanism, morphological and structural changes, oxidative stress, photosynthesis, autophagic flux, and global gene expression were investigated. Biochemical analyses showed that the YX04 supernatant induced reactive oxygen species (ROS) overproduction, which caused lipid peroxidation and malondialdehyde (MDA) accumulation in *P. globosa*. Transmission electron microscopy (TEM) observation and the significant decrease in both maximum photochemical quantum yield (Fv/Fm) and relative electron transfer rate (rETR) indicated damage to thylakoid membranes and destruction of photosynthetic system function. Immunofluorescence, immunoblot, and TEM analyses indicated that cellular damage caused autophagosome formation and triggered large-scale autophagic flux in *P. globosa*. Transcriptome analysis revealed many *P. globosa* genes that were differentially expressed in response to YX04 stress, most of which were involved in photosynthesis, respiration, cytoskeleton, microtubule, and autophagosome formation and fusion processes, which may trigger autophagic cell death. In addition to *P. globosa*, the YX04 supernatant showed high algicidal activity against Thalassiosira pseudonana, Thalassiosira weissflogii, Skeletonema costatum, Heterosigma akashiwo, and Prorocentrum donghaiense. This study highlights multiple mechanisms underlying YX04 supernatant toxicity toward *P. globosa* and its potential for controlling the occurrence of harmful algal blooms.

**IMPORTANCE** *P. globosa* is one of the most notorious harmful algal bloom (HAB)-causing species, which can secrete hemolytic toxins, frequently cause serious ecological pollution, and pose a health hazard to animals and humans. Hence, screening for bacteria with high algicidal activity against *P. globosa* and studies on the algicidal characteristics and mechanism will contribute to providing an ecofriendly microorganism-controlling agent for preventing the occurrence of algal blooms and reducing the harm of algal blooms to the environment. Our study first reported the algicidal characteristic and mechanism of *Microbulbifer* sp. YX04 against *P. globosa* and demonstrated that *P. globosa* shows different response mechanisms, including movement ability, antioxidative systems, photosynthetic systems, gene expression, and cell death mode, to adapt to the adverse environment when algicidal compounds are present.

## INTRODUCTION

Over the past several decades, harmful algal blooms (HABs) have frequently occurred and threatened most coastal countries, as phytoplankton abnormally proliferate or aggregate and produce potent toxins, thereby discoloring and damaging seawater (1). Phaeocystis globosa, one of the most notorious HAB-causing species, can secrete hemolytic toxins and produce fetid foam with nauseating smells, thereby substantially damaging marine ecosystems and tourism industries, threatening public health and jeopardizing the safe operation of nuclear power plants by blocking the water intake of cold source systems (2–4).

Many physical (clay, UV irradiation, etc.) and chemical (herbicides, metals, photosensitizers, etc.) methods have been used to mitigate and control HABs; however, these methods pose the risks of secondary ecological/environmental problems and are difficult to implement (5, 6). In the marine environment, zooplankton, bacteria, and viruses living with phytoplankton are known as predators, competitors, and inhibitors and influence the dynamics of phytoplankton populations (7). Taking advantage of the natural relationship between microorganisms and phytoplankton to control or reduce the adverse effects of HABs has become a research hot spot due to the environmental friendliness of this method (6, 8). Numerous bacteria are reported to be capable of killing HAB species (6). A few algicidal bacteria lyse algal cells by directly attaching to each other, which is termed the direct algicidal mode (9, 10). A variety of algicidal bacteria produce and release metabolic substances to kill algal cells, which is considered to be an indirect algicidal mode. Different kinds of algicidal compounds, such as pigments, proteins, rhamnolipids, and biosurfactants, have been identified (11–17). The metabolites of the strains *Bacillus* sp. LP-10 (18) and *Streptomyces* sp. JS01 (19) exhibited strong algal-lysing activities against P. globosa. Prodigiosin produced by *Hahella* sp. KA22 was shown to induce a reactive oxygen species (ROS) burst in *P. globosa*, which could result in severe oxidative damage and algal cell photosynthetic destructions (17). Three compounds produced by *Bacillus* sp. strain B1, urocanic acid, L-histidine, and N-acetylhistamine, showed remarkably high algicidal activity against *P. globosa* when applied in combination (20). The antialgal compound luteolin-7-O-glucuronide inhibited the growth of *P. globosa* by disturbing the tricarboxylic acid (TCA) cycle of algal cells (4).

Algicidal substances are usually reported to induce excessive ROS production in algal cells (11, 14, 16–18, 21, 22). To avoid oxidative damage, algal cells have developed diverse protection mechanisms, such as antioxidant enzymes superoxide dismutase (SOD), catalase (CAT), and peroxidase (POD) and the nonenzymatic antioxidant glutathione (GSH). Although algal cells have been shown to enhance antioxidant system activity, excessive ROS still initiate lipid peroxidation, which disrupts cellular membrane integrity and damages the photosynthetic system. Moreover, oxidative stress significantly decreases the contents of photosynthetic pigments, proteins, and carbohydrates and downregulates the expression of photosynthesis- and respiratory-related genes in algal cells, which decreases the photosynthetic efficiency and capacity and disrupts the respiratory system, thereby increasing ROS production again (5, 11, 17, 18, 20–22). Therefore, the antioxidative response of algal cells does not ultimately prevent cell death.

The mechanism of algal cell death not only affects the adaptation of the algal population to adverse environmental conditions but also has important significance for determining the flow of photosynthetically fixed organic matter and even for regulating the biogeochemical cycles of aquatic ecosystems (23). Therefore, determining the death modes of algal cells encountering abiotic or biotic environmental stresses is important. Caspase-mediated apoptotic cell death has been documented in Dunaliella tertiolecta, Dunaliella viridis, Dunaliella salina, Micrasterias denticulata, and Chlorella saccharophila under conditions of prolonged darkness (24), senescence (25), salt (26, 27), heat (25, 28), carbon (29), and nitrogen (25) stress. Autophagy, which involves autophagy-related genes (ATGs) and the formation of autophagosomes, was verified as a death mode in Chlamydomonas reinhardtii and *Micrasterias denticulata* under the conditions of nitrogen limitation (30) and carbon starvation (31). Prodigiosin was shown to induce necrotic-like or apoptotic-like cell death in Microcystis aeruginosa (32). Viral infection was shown to trigger autophagosome formation and caspase activity-dependent apoptosis in Emiliania huxleyi during the lytic phase (33–35), which led to the demise of large-scale E. huxleyi blooms (36).

Although a variety of algicidal bacteria against *P. globosa* have been identified and their algicidal effects have been evaluated, the underlying programmed death mode and molecular mechanism of *P. globosa* remain unclear. In this study, the bacterial strain YX04, which showed high algicidal activity against *P. globosa*, was isolated and identified. To explore its algicidal effect, the algicidal mode, stability of algicidal activity, and algicidal spectrum of strain YX04 were investigated. To explore the algicidal mechanism of strain YX04 and the death mode of *P. globosa*, we (i) observed the morphological and structural changes in *P. globosa* using scanning electron microscopy (SEM) and transmission electron microscopy (TEM), (ii) investigated the oxidative stress and antioxidant responses, (iii) examined photosynthetic damage using fast chlorophyll fluorescence on a pulse-amplitude modulation fluorometer, (iv) compared gene expression differences by transcriptome analysis, and finally, (v) detected autophagic flux in *P. globosa* by immunofluorescence and immunoblotting techniques.

## RESULTS

### Algae-killing activity and identification of strain YX04.

**FIG 1:**
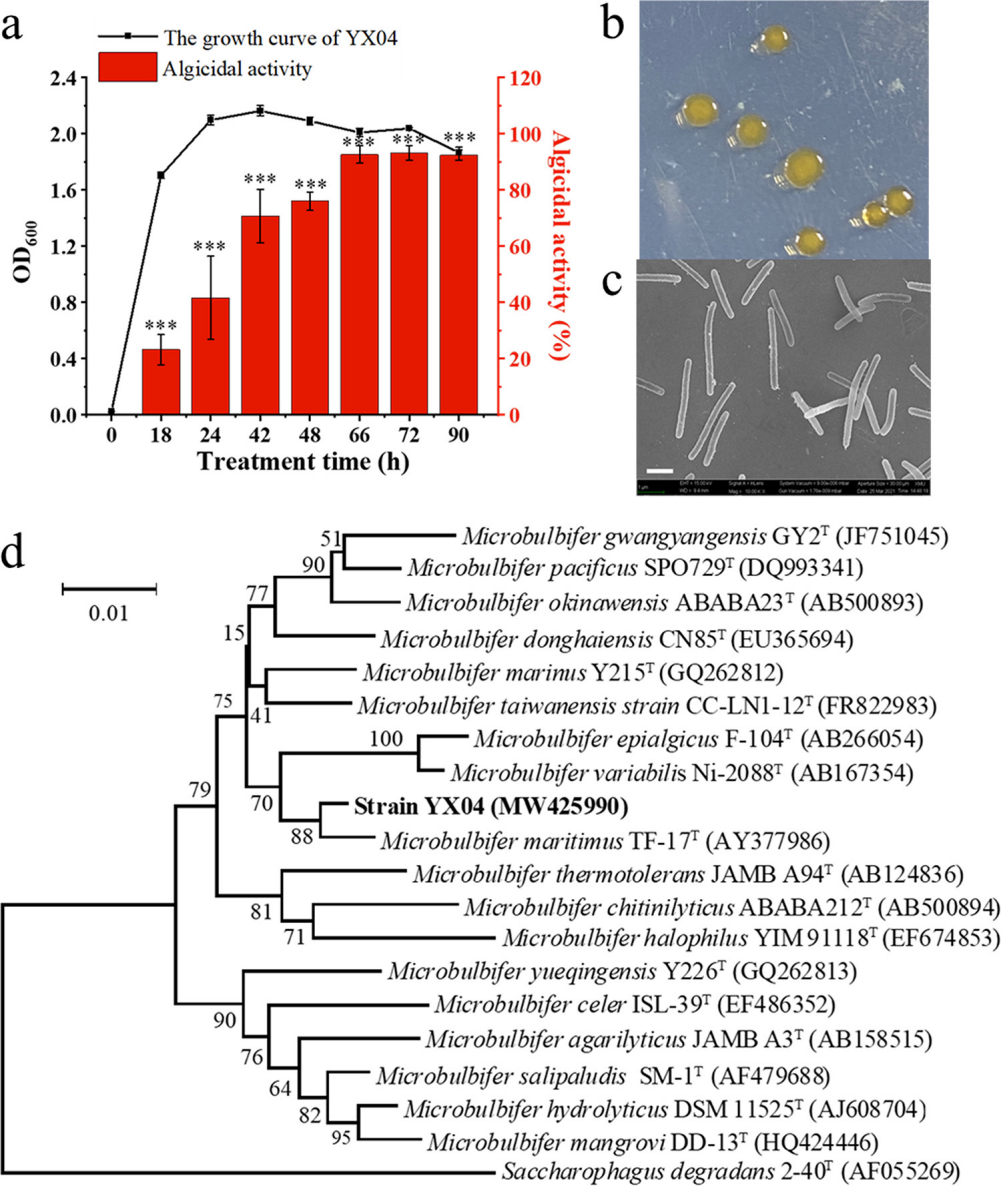
Isolation and identification of the algicidal bacterium YX04. (a) Algicidal activity at different growth stages. All the error bars indicate the standard errors (SEs) of three biological replicates. *, *P < *0.05; **, *P < *0.01; ***, *P < *0.001 compared with the 0 h growth of the YX04 supernatant. (b) YX04 colonies growing on the 2216E plate. (c) Morphology of strain YX04 under SEM. (d) Phylogenetic positions of strain YX04.

Strain YX04, isolated at the Yunxiao Mangrove National Nature Reserve in Fujian Province, showed strong algicidal activity against *P. globosa* in the exponential and declining phases. The algae-killing activity of the YX04 culture increased as the culture time was extended, peaking at 93.2% in the declining phase (Fig. 1a). The algicidal substances of YX04 accumulated throughout the growth process, peaking at the declining phase. YX04 cells grew yellow colonies with serrated borders on 2216E agar plates after incubation for 48 h (Fig. 1b). SEM showed that strain YX04 was rod-shaped and slightly curved (Fig. 1c). PCR amplification of the 16S rRNA gene (1,403 bp, GenBank accession number: MW425990) and sequencing revealed that the strain YX04 sequence was most similar (99.36%) to Microbulbifer maritimus TF-17 (Fig. 1d). Therefore, strain YX04 was determined to belong to the *Microbulbifer* genus and was named *Microbulbifer* sp. YX04. The *Microbulbifer* genus has never been reported to have algicidal activity.

### Algicidal characterization of strain YX04.

**FIG 2:**
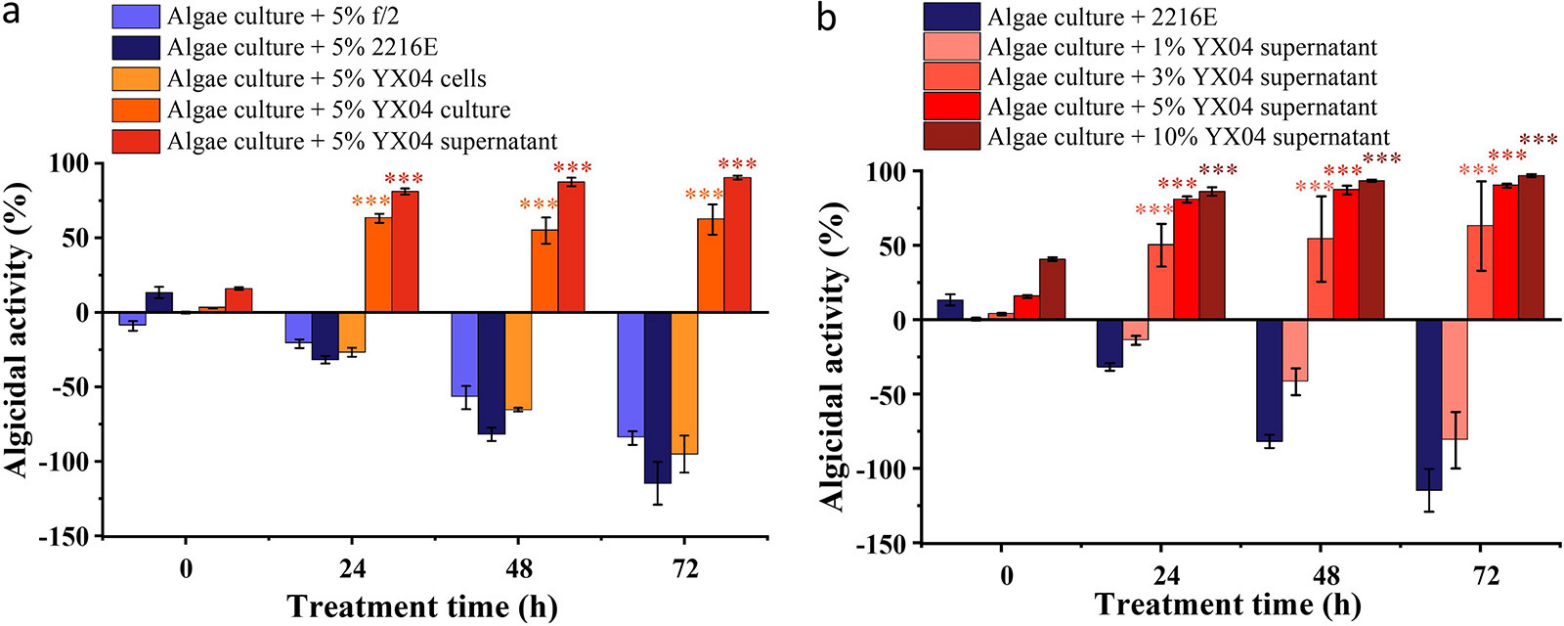
Algicidal mode and activities of strain YX04 against *P. globosa*. (a) Algicidal activities of different fractions of YX04 culture. (b) Algicidal rate of different concentrations of the YX04 supernatant. All error bars indicate the SEs of three biological replicates. ***, *P < *0.001.

To investigate the algicidal mode of strain YX04, different fractions of strain YX04 cultures, including bacterial cells and the cell-free supernatant, were added to the algal cultures at a concentration of 5% (vol/vol) to assess their algicidal activities. As shown in Fig. 2a, the bacterial culture and cell-free supernatant of YX04 exhibited significant algicidal activity, while the bacterial cells did not influence the growth of *P. globosa*. Because the YX04 supernatant had higher activity than the bacterial culture, strain YX04 killed algal cells, probably by secreting active compounds into the supernatant. Figure 2b shows that the algicidal activity of the YX04 supernatant increased in a concentration- and time-dependent manner. Within 24 h of treatment, the 5% and 10% supernatant treatment groups showed higher algalytic activities (81% and 86%) than the 3% treatment group (50%), while the 1% supernatant group only inhibited the growth of *P*. *globosa* and did not kill algal cells. The algicidal activities of the 3%, 5%, and 10% supernatant groups were slightly increased and reached 55 to 63%, 87 to 90%, and 93 to 97% after treatment for 48 and 72 h, respectively. These results indicated that the algicidal activity of the YX04 supernatant was concentration dependent. Because the 5% YX04 supernatant group exhibited an algicidal rate higher than 90%, the 5% dose was selected for subsequent studies.

To determine the algicidal characteristics of the YX04 supernatant, its thermal and pH stability were investigated by exposure to different temperatures and pH values for 2 h. As shown in Fig. S1, the algicidal activities of the YX04 supernatant were stable after exposure to a wide range of pH values (4 to 12) and temperatures (−80 to 100°C) for 2 h. This result indicated that algicidal substances were insensitive to acidic (above a pH of 4) and alkaline (below a pH of 12) environments and had thermal stability. The stability of the algicidal activity suggested that the YX04 supernatant could be temporarily stored in various kinds of adverse environmental conditions.

To determine the algae-killing spectrum of strain YX04, 11 different algal species were tested. As shown in Table S1, YX04 exhibited strong algicidal effects against Thalassiosira pseudonana, Thalassiosira weissflogii, and Skeletonema costatum in Bacillariophyceae, against Heterosigma akashiwo in Xanthophyceae, against Prorocentrum donghaiense in Pyrrophyta, and against *Phaeocystis globosa* in Chrysophyta. However, YX04 had no alga-killing effect on any of the tested algal species in Chlorophyta.

### Strain YX04 induced oxidative stress in *P. globosa*.

**FIG 3:**
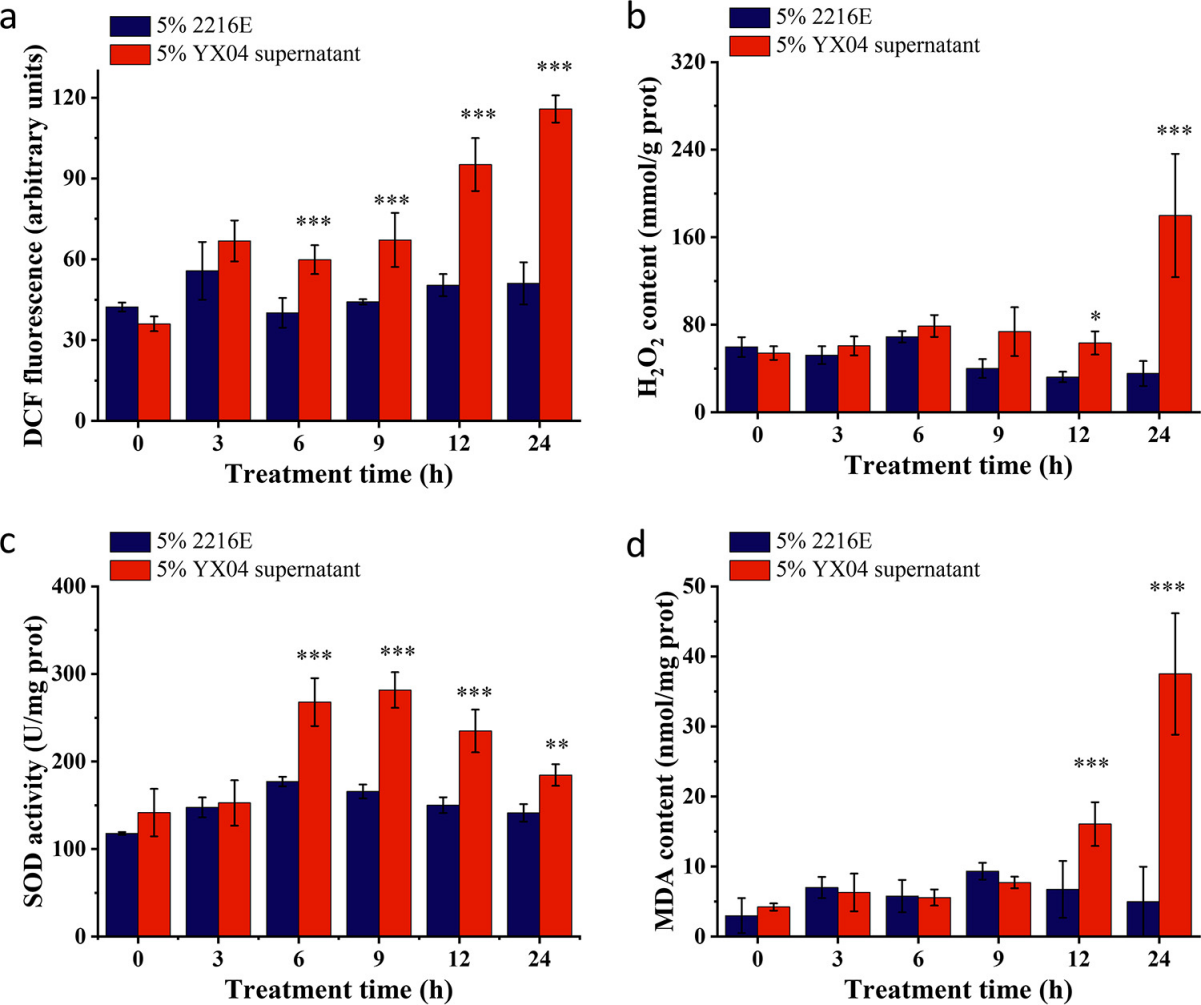
Effects of the YX04 supernatant on oxidative stress and the antioxidant defense system in *P. globosa*. (a) The ROS content, (b) H_2_O_2_ content, (c) activity of the antioxidant SOD, and (d) MDA content. All error bars indicate the standard error of three replicates. *, *P < *0.05 **, *P < *0.01; ***, *P < *0.001 compared with the control group.

Algicidal bacteria usually cause algal cell death by inducing excessive ROS production. To investigate the oxidative stress caused by strain YX04, we detected the ROS and H_2_O_2_ contents in *P. globosa*. As shown in Fig. 3a, the dichlorodihydrofluorescein diacetate (DCFH-DA) fluorescence intensity was significantly increased in algal cells after treatment with the 5% YX04 supernatant for 6 h, with the peak fluorescence value at 24 h being 2.03-fold higher than that in the control group, indicating a marked increase in the ROS content after 6 h of treatment. In accordance with the increased ROS contents, the H_2_O_2_ contents were also significantly increased after treatment for 24 h, peaking at 179.9 mmol/g prot (5.07-fold higher than those of the control group) (Fig. 3b). The enzymatic activity of SOD in the YX04 treatment group continued to increase after 6 h of treatment and peaked at 281.6 U/mg prot after treatment for 9 h (1.7-fold higher than that of the control) (Fig. 3c). This result suggested that the massive accumulation of H_2_O_2_ was enhanced by increased SOD activity.

The burst of ROS often initiates lipid peroxidation (37) and results in malondialdehyde (MDA) accumulation and membrane lipid destruction. The increased MDA content reflects cellular oxidative damage under adverse conditions (38). As shown in Fig. 3d, the MDA content was markedly enhanced by 2.39- and 7.5-fold compared with that in the control group after treatment with the YX04 supernatant for 12 and 24 h, respectively, suggesting that lipid peroxidation was strongly induced and thus reflected severe oxidative damage in the cell membrane system.

### Transcriptome analysis of *P. globosa* treated with the YX04 supernatant.

To reveal the cellular responses and molecular mechanisms associated with the oxidative stress and cell fate decisions of *P. globosa* in response to the algicidal strain YX04, we assessed the changes in global gene expression in *P. globosa* after treatment with 5% YX04 supernatant for 6, 12, and 24 h by performing transcriptome analysis. In total, 6.78 Gb of clean data per sample was obtained by transcriptome sequencing, yielding more than 95% Q_30_ bases. All clean data were assembled *de novo*, producing 69,230 unigenes and 94,211 transcriptions. Among them, 11,312 unigenes were subjected to Gene Ontology (GO) annotation and divided into 3 groups: biological process (BP), cellular component (CC), and molecular function (MF) (Fig. S2); *Arabidopsis* and Chlamydomonas reinhardtii served as references.

**FIG 4:**
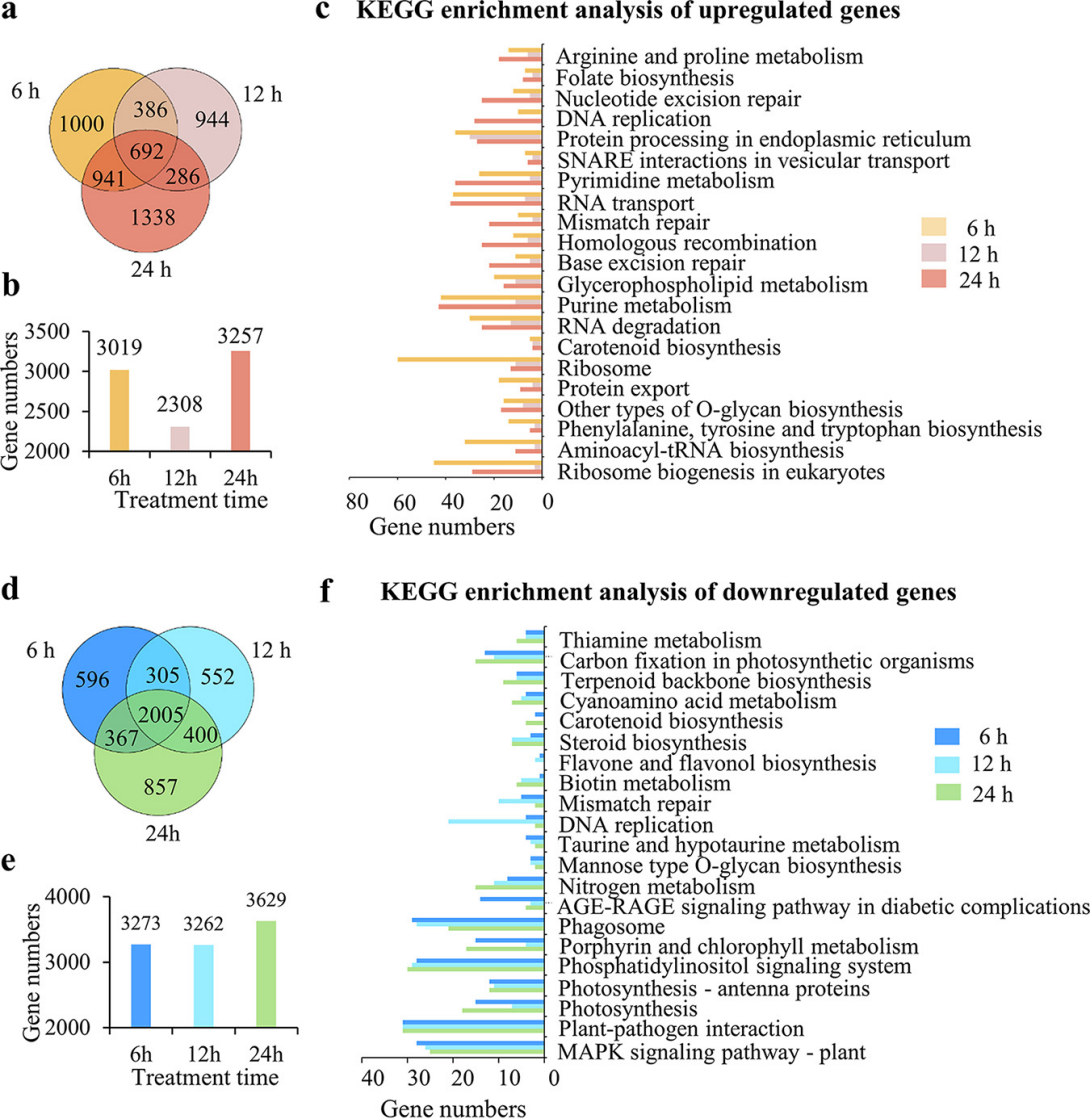
Differentially expressed genes in *P. globosa* treated with 5% YX04 supernatant for different times. (a) Venn diagram of the upregulated genes after *P. globosa* treatment for different amounts of time. (b) Numbers of upregulated genes in *P. globosa* treatment for different amounts of time. (c) Enriched KEGG pathways of the upregulated genes. (d) Venn diagram of the downregulated genes. (e) Numbers of downregulated genes in *P. globosa* at different treatment time points. (f) Enriched KEGG pathways of the downregulated genes.

To further elucidate the molecular response of *P. globosa* to the YX04 supernatant, the significantly upregulated and downregulated genes in the YX04 treatment groups were used to construct a large gene set, and KEGG pathway analysis of differentially expressed genes (DEGs) was then conducted. Compared with those in the control group, 3,019, 2,308, and 3,257 genes were upregulated in the treatment groups after 6, 12, and 24 h of treatment, respectively. Among them, 692 genes were upregulated at all three time points (Fig. 4a and b). In the upregulated gene set, 60 pathways were enriched by KEGG. The top 25 terms for significantly enriched pathways (Fig. 4c) included ribosome, purine, and pyrimidine metabolism, RNA transport and degradation, protein processing in the endoplasmic reticulum (ER), peroxisomes, protein export, mismatch repair, nucleotide excision repair, DNA repair, glycerophospholipid and glycerolipid metabolism, and SNARE interactions in vesicular transport. While more genes were found to be downregulated than upregulated, 3,273, 3,262, and 3,629 genes were downregulated after treatment for 6, 12, and 24 h, respectively, compared with those in the control group. A total of 2,005 genes were downregulated in all treatment groups (Fig. 4d and e). In the downregulated gene set, 85 pathways were enriched by KEGG. The top 21 significantly enriched pathways included the MAPK signaling pathway, photosynthesis, photosynthesis-antenna proteins, porphyrin and chlorophyll metabolism, carbon fixation in photosynthetic organisms, carotenoid biosynthesis, nitrogen metabolism, and DNA replication (Fig. 4f). Among these pathways, photosynthesis appeared 5 times, indicating the marked inhibition of photosynthesis in *P. globosa* after treatment with the YX04 supernatant. To elucidate pivotal mechanisms of *P. globosa* death triggered by YX04, gene expression changes combined with the morphological, physical, and chemical properties are described in detail in a separate part of this paper to elaborate on the mode and process of algal death.

### Strain YX04 induced *P. globosa* photosynthetic system damage.

**FIG 5:**
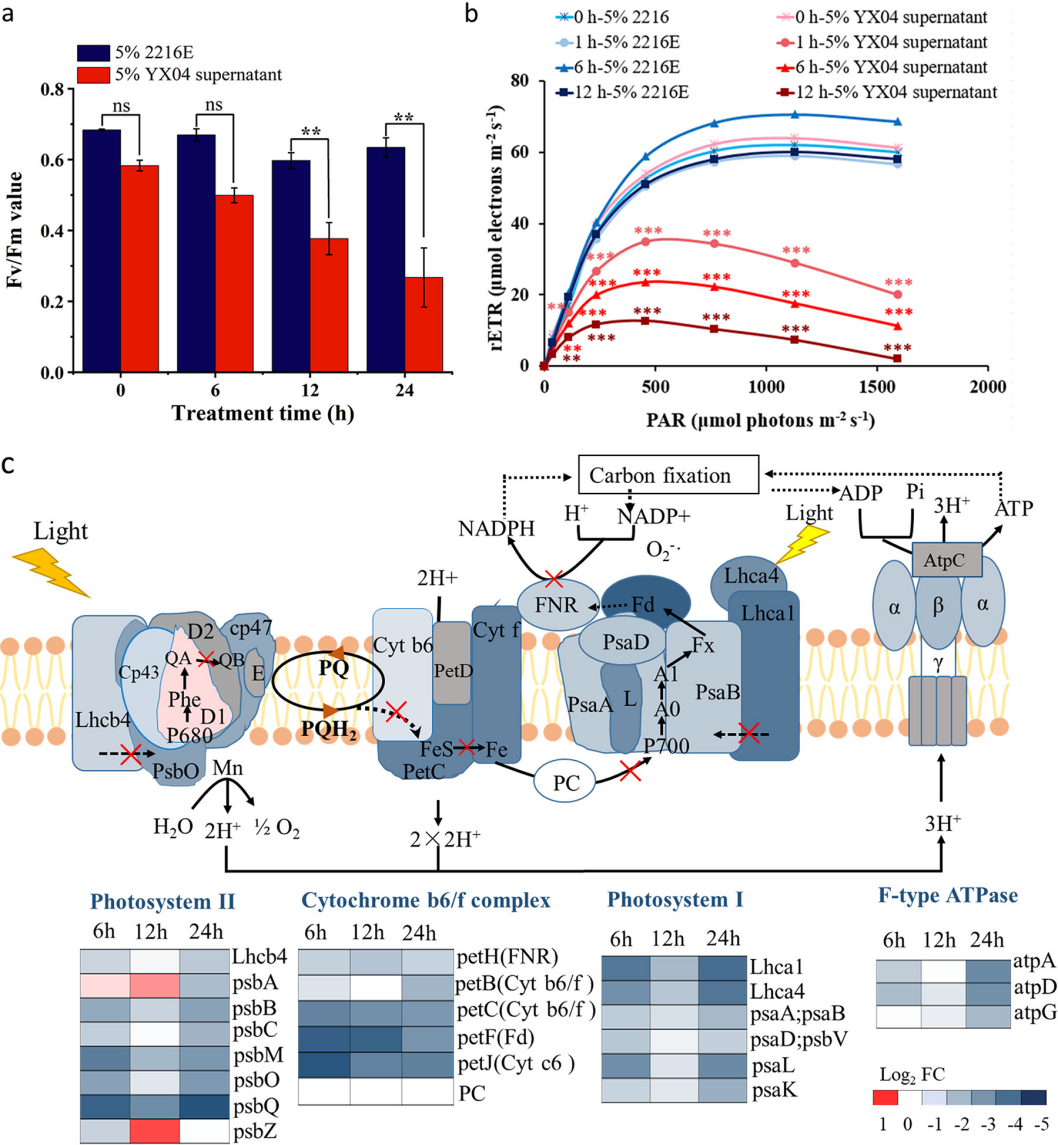
Effects of the YX04 supernatant on the photosynthetic system of *P. globosa*. (a) The maximum photochemical quantum yield, (b) photosynthetic electron transport rate, and (c) differentially expressed genes in the photosynthetic system after treatment with the 5% YX04 supernatant for 6, 12, and 24 h (adjusted *P* value of <0.05 and |log_2_FC| of <−1). All error bars indicate the SEs of three biological replicates. **, *P < *0.01 compared with the control group.

Algal cells capture light energy, which is converted to chemical energy by photosynthesis. The Fv/Fm ratio represents the maximum quantum yield of photosystem (PS) II, and rETR represents the photosynthetic electron transport rate (39). As shown in Fig. 5a, the Fv/Fm values of the control group were stable between 0.59 and 0.68, while the values gradually decreased from 0.583 to 0.267 during 24 h of exposure. The rETR values of the treatment groups were markedly decreased by 65% and 84% after treatment for 6 and 12 h, respectively, while the maximum rETR values of the control were stable at approximately 60 μmol electron m^−2^ s^−1^ (Fig. 5b). The results indicated damage to photosynthetic electron transport. Photosynthetic electron transport depends on the membrane system being intact. Fig. 3d shows strong lipid peroxidation in YX04-treated algal cells. Lipid peroxidation could disrupt the cellular membrane system. Thus, we concluded that the damage to photosynthetic electron transport was due to the oxidative destruction of thylakoid membranes.

Transcriptome analysis also indicated that most of the primary genes related to photochemical reactions, photosynthetic electron transport, and photophosphorylation, including PS II genes (*Lhcb4*, *psb*B, *psb*C, *psb*M, *psb*O, *psb*Q, *psb*27), cytochrome (Cyt) b6/f complex genes (*pet*B, *pet*C, *pet*J), PS I genes (*Lhca1*, *Lhca4*, *psa*A/*psa*B, *psa*D/*psa*V, *psa*K, *psa*L), photosynthetic electron transport genes (*pet*F, *pet*H), and F-type ATPase genes (*atp*A, *atp*D, *atp*G), were markedly downregulated after treatment with the 5% YX04 supernatant (Fig. 5c). This result indicated that the YX04 supernatant seriously damaged the photosynthetic system of *P. globosa* not only by damaging the membrane system but also by downregulating photosynthetic gene expression to hinder photosynthetic system repair.

### Transcriptome analysis revealed mitochondrial dysfunction and oxidative stress accumulation in *P. globosa* triggered by strain YX04.

**FIG 6:**
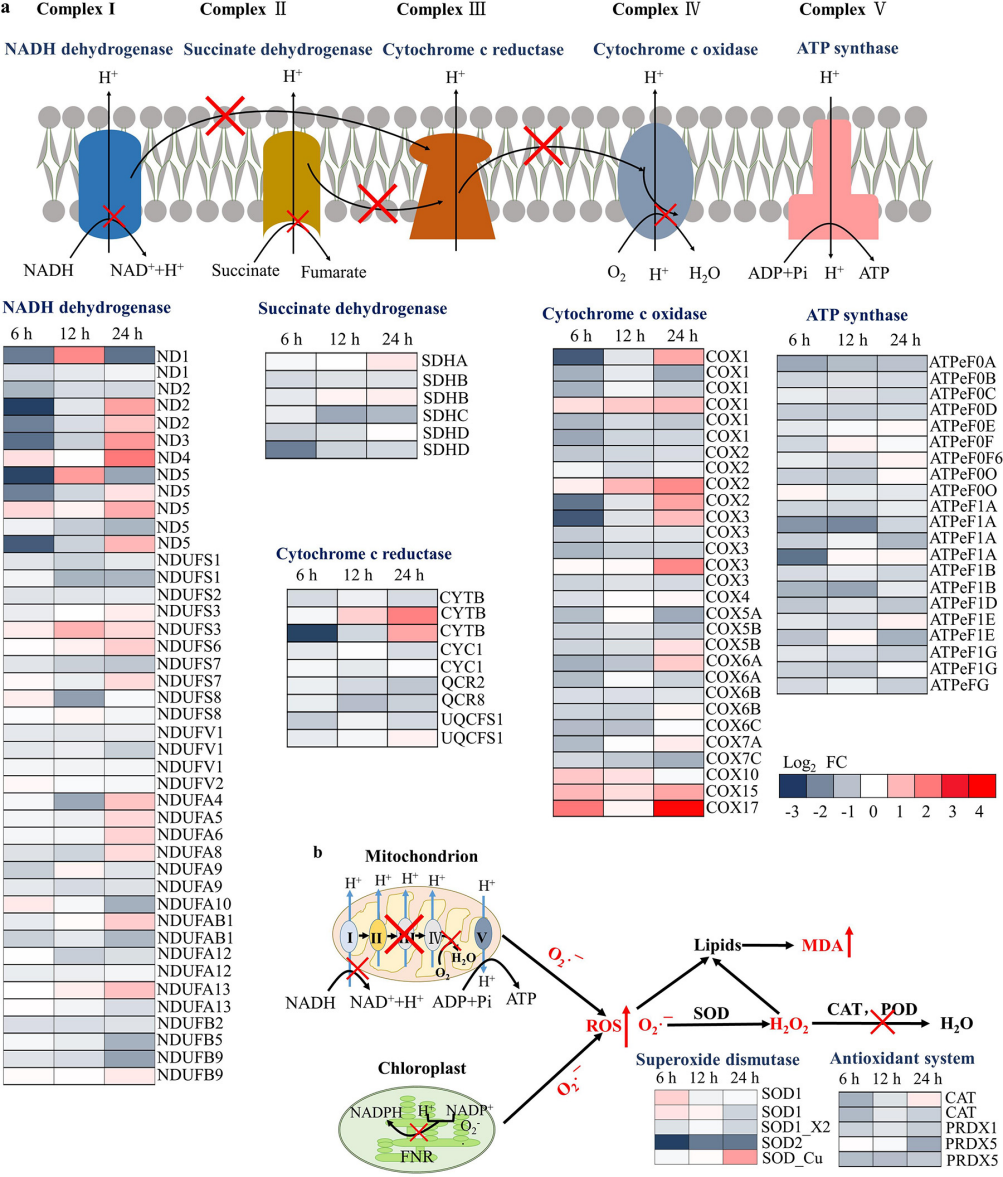
Differentially expressed genes related to ROS production and the antioxidant defense system in *P. globosa* after treatment with the YX04 supernatant. Differentially expressed genes in the respiratory electron transport chain (a) and in the antioxidant defense system (b) after treatment with the 5% YX04 supernatant for 6, 12, and 24 h (adjusted *P* value of <0.05 and |log_2_FC| of <−1).

According to Fig. 3, the YX04 supernatant markedly increased ROS and H_2_O_2_ contents in algae cells. It is well known that the formation of ROS occurs either in the mitochondria during electron transport from highly reducing components to molecular oxygen or in the chloroplast during the light reaction of photosynthesis and in the membrane-bound enzyme complex NADPH oxidase (40, 41). As shown in Fig. 6a, most genes related to the respiratory electron transport chain in mitochondria, including complex I NADH dehydrogenase (ND1-ND5, NDUFS, NDUFV, NDUFA, NDUFAB, and NDUFB), complex II succinate dehydrogenase (SDHA, SDHB SDHC, and SDHD), complex III cytochrome c reductase (CYTB, CYC1, QCR2, QCR6, and UQCFS1), complex IV cytochrome c oxidase (COX1-COX7), and ATP synthase (ATPeF_0_A to ATPeF_0_F, ATPeF_0_O, ATPeF_1_A, ATPeF_1_B, ATPeF_1_D, ATPeF_1_E, and ATPeF_1_G), were downregulated in *P. globosa* after treatment with the YX04 supernatant for 6 h, indicating dysfunction of the respiratory chain. The dysfunction of both respiratory and photosynthetic electron transport (Fig. 5) resulted in the overproduction of superoxide anion radicals (O_2_^•−^). The superoxide anion radicals (O_2_^•−^) can be further reduced to H_2_O_2_ and hydroxyl radicals (HO^•^), which is catalyzed by various types of superoxide dismutase (SOD) and peroxidase family enzymes, including peroxidase (POD) and catalase (CAT).

Transcriptome analysis (Fig. 6b) showed that the expression of several genes of the SOD family (SOD1 and SOD-Cu) was upregulated after treatment with the YX04 supernatant. SOD2 (mitochondrial SOD-Mn) gene expression was continuously downregulated. This result indicated that different types of SOD played antioxidant roles at different times after the treatment, and excessive O_2_^•−^ in the mitochondria was not reduced, which resulted in the accumulation of O_2_^•−^ and the formation of oxidative stress. The expression of peroxidase genes (PRDX) and catalase genes (CAT) was markedly downregulated after treatment with YX04 supernatant (Fig. 6b), which resulted in the decrease of H_2_O_2_ reduction. The accumulation of H_2_O_2_ further strengthened the oxidative stress in *P. globosa*. These transcriptome analysis results related to ROS and H_2_O_2_ production as well as SOD activity were in accordance with their changes in Fig. 3.

### Strain YX04 induced external morphological changes in *P. globosa*.

**FIG 7:**
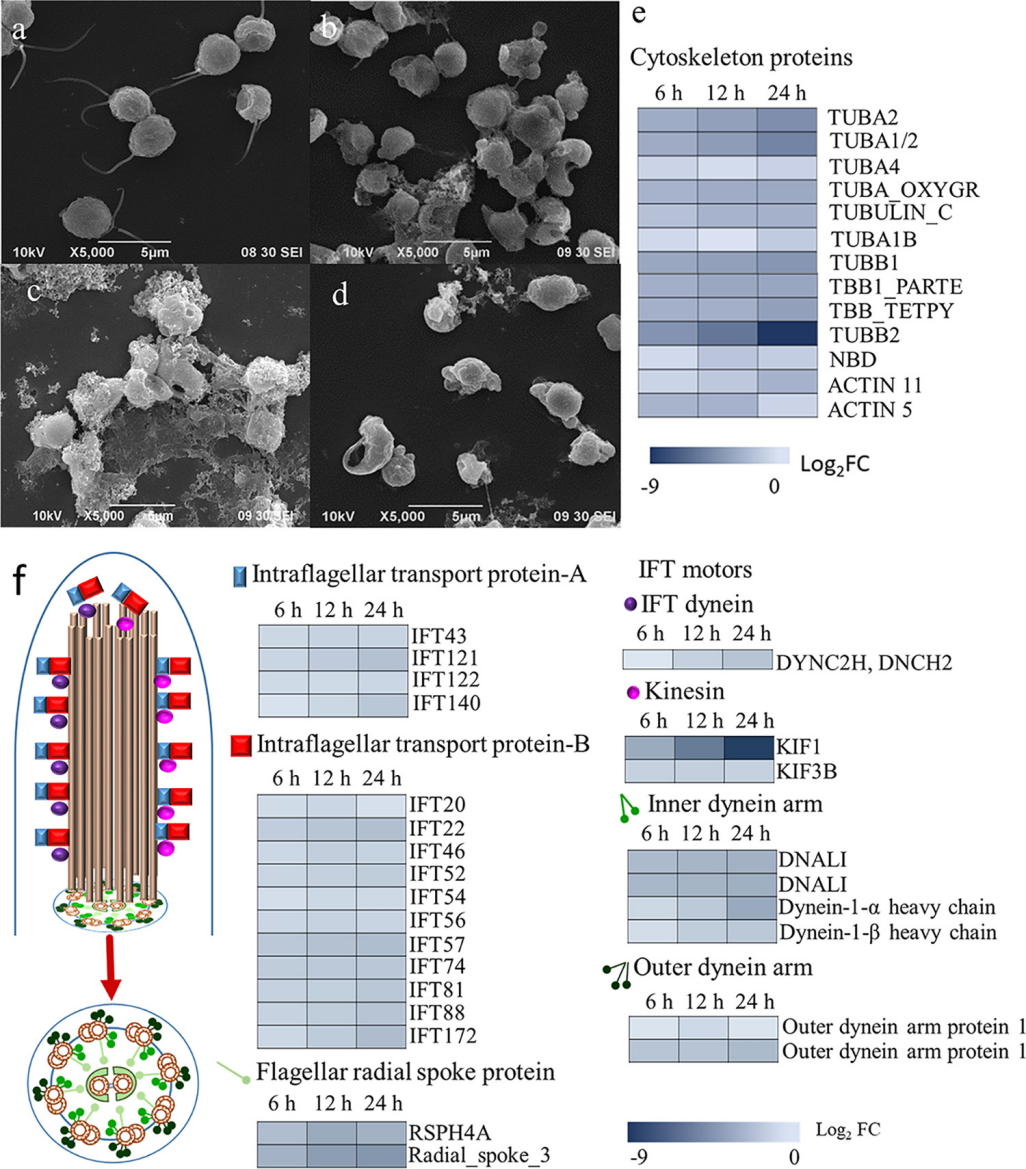
Morphological changes of *P. globosa* treated with 5% YX04 supernatant. SEM images of algal cells treated with 5% 2216E broth (a) and 5% YX04 supernatant for 6 h (b), 12 h (c), and 24 h (d). (e) Heat map of differential expression of genes related to cytoskeleton. (f) Flagellum structure and heat maps of differential expression of genes related to flagellum assembly and motility.

The external morphological changes in *P. globosa* treated with the 5% YX04 supernatant were observed using SEM. Healthy algal cells had two flagellar lengths (4.5 to 5.5 μm) with full spheres (Fig. 7a). Treatment with the YX04 supernatant for 6 h caused flagella shortening or disappearance (Fig. 7b). After 12 h of treatment, more cells produced bubbles, exhibited deformity, and no longer maintained a round shape (Fig. 7c and d).

Transcriptome analysis showed that 13 genes related to the cytoskeleton in the YX04-treated group were significantly downregulated compared to those in the control group (Fig. 7e), indicating that the YX04 supernatant destroyed the cytoskeletal structure and changed the morphology of algal cells. In addition to cytoskeleton-related genes, 26 genes encoding proteins related to motility, including intraflagellar transport protein A (IFT-A), IFT-B, flagellar radial spoke proteins, IFT dynein, kinesin, inner dynein arm, and outer dynein arm, were significantly downregulated in the YX04-treated group (Fig. 7f), suggesting that the YX04 supernatant induced the loss of algal cell motor ability. These transcriptome analysis results were in accordance with the morphological changes in algal cells determined by SEM.

### Strain YX04 induced autophagic *P. globosa* cell death.

**FIG 8:**
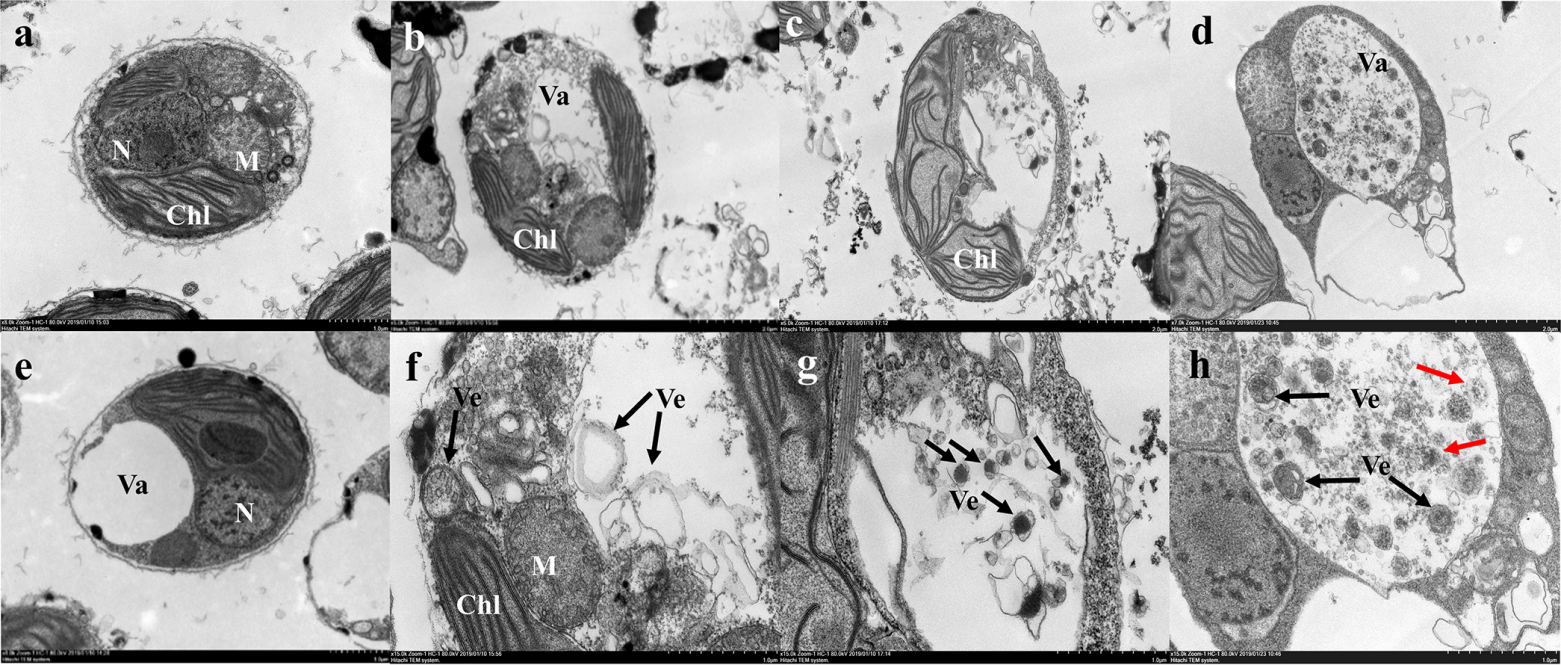
Structural changes in *P. globosa* treated with the 5% YX04 supernatant. TEM images of algal cells treated with 2216E (a and e) and the YX04 supernatant for 6 h (b and f), 12 h (c and g), and 24 h (d and h). Scale = 1 μm (a to e) and 2 μm (f to h). Chl, chloroplast; M, mitochondria; N, nucleus; Va, vacuole, Ve, vesicles. Red arrow: degraded vesicles.

Changes in the internal structure of algal cells were observed using TEM. The controls were healthy and exhibited intact plasma membranes, tightly stacked lamellar chloroplast structures, intact mitochondria and nuclei, and clear vacuoles (Fig. 8a and e). Mitochondrial ridges were not obvious and grana lamellae were sparse after treatment with the YX04 supernatant from 6 h to 24 h (Fig. 8b to d). Numerous vesicles were observed in algal cells treated with the YX04 supernatant, and most had double-layer membranes (Fig. 8f and g). After 24 h of treatment, the vacuole was markedly enlarged, with the diameter reaching 1.5 to 2.5 by 3 to 4 μm, and was full of vesicles (Fig. 8d and h, black arrow). In the vacuoles, some vesicles showed distinct membranes (red arrows) and were thus most likely degraded. All of the vesicles with double-layer or indistinct membranes were produced in the cytoplasm and degraded in the vacuole, which is very similar to the production and degradation of autophagosomes in plants. LysoTracker Green, an acidic dye used to label vacuoles, lysosomes, and autophagosomes, was utilized to investigate the possible function of the vesicles. The results showed a markedly increased LysoTracker Green fluorescence signal in *P. globosa* after 3 h of treatment, and the fluorescence intensity increased as the treatment time was extended, indicating acidic vesicle production in the treated cells (Fig. S3a). Flow cytometry detection revealed an increasing number of positive cells labeled with LysoTracker Green over time (Fig. S3b and c). TEM observations and analysis of acidic vesicle production suggested the formation of autophagosomes in YX04-treated *P. globosa*.

**FIG 9:**
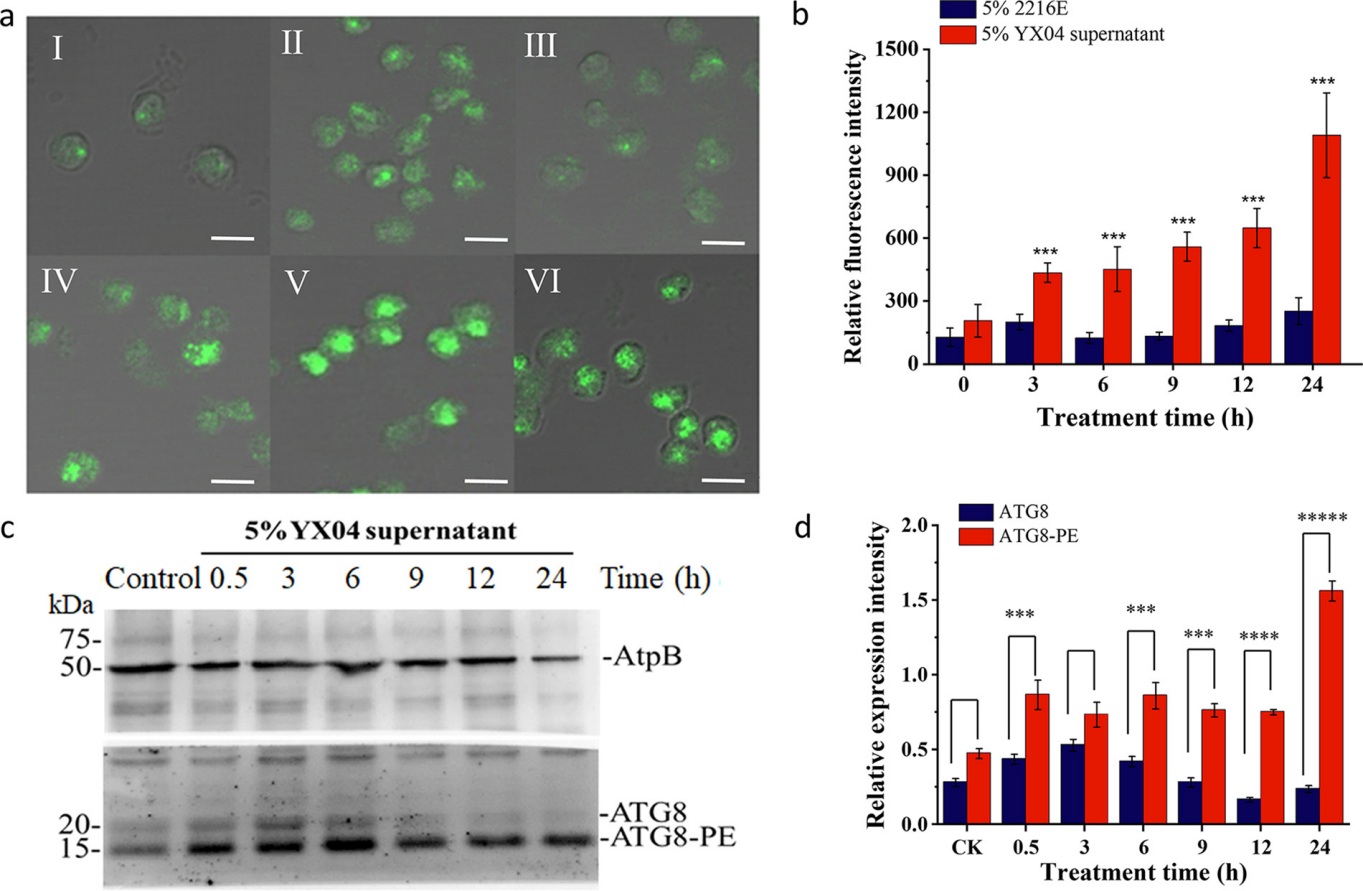
Detection of the autophagic flux in *P. globosa* cells treated with 5% YX04 supernatant. (a) Immunolocalization of ATG8 in *P. globosa* cells treated with 5% YX04 supernatant at 3 (II), 6 (III), 9 (IV), 12 (V), and 24 h (VI) and with the control 5% 2216E broth for 24 h (I), scale bar = 5 μm. (b) Relative immunofluorescence intensity of panel a. Thirty cells were selected in each sample to calculate the mean value; the error bars indicate the standard errors of 30 cells. (c) Western blotting (WB) of ATG8 protein expression in algal cells treated with 5% YX04 supernatant for different amounts of time. Anti-AtpB antibodies were used as the loading control. (d) The relative intensities of the WB bands were analyzed using ImageJ software. The *y* coordinate indicates the ratio of the gray value of the ATG8 band to the gray value of the band corresponding to the internal reference protein AtpB. Different sample collection time points are labeled on the *x* coordinate. *, *P < *0.05; **, *P < *0.01; ***, *P < *0.001 compared with the control.

**FIG 10:**
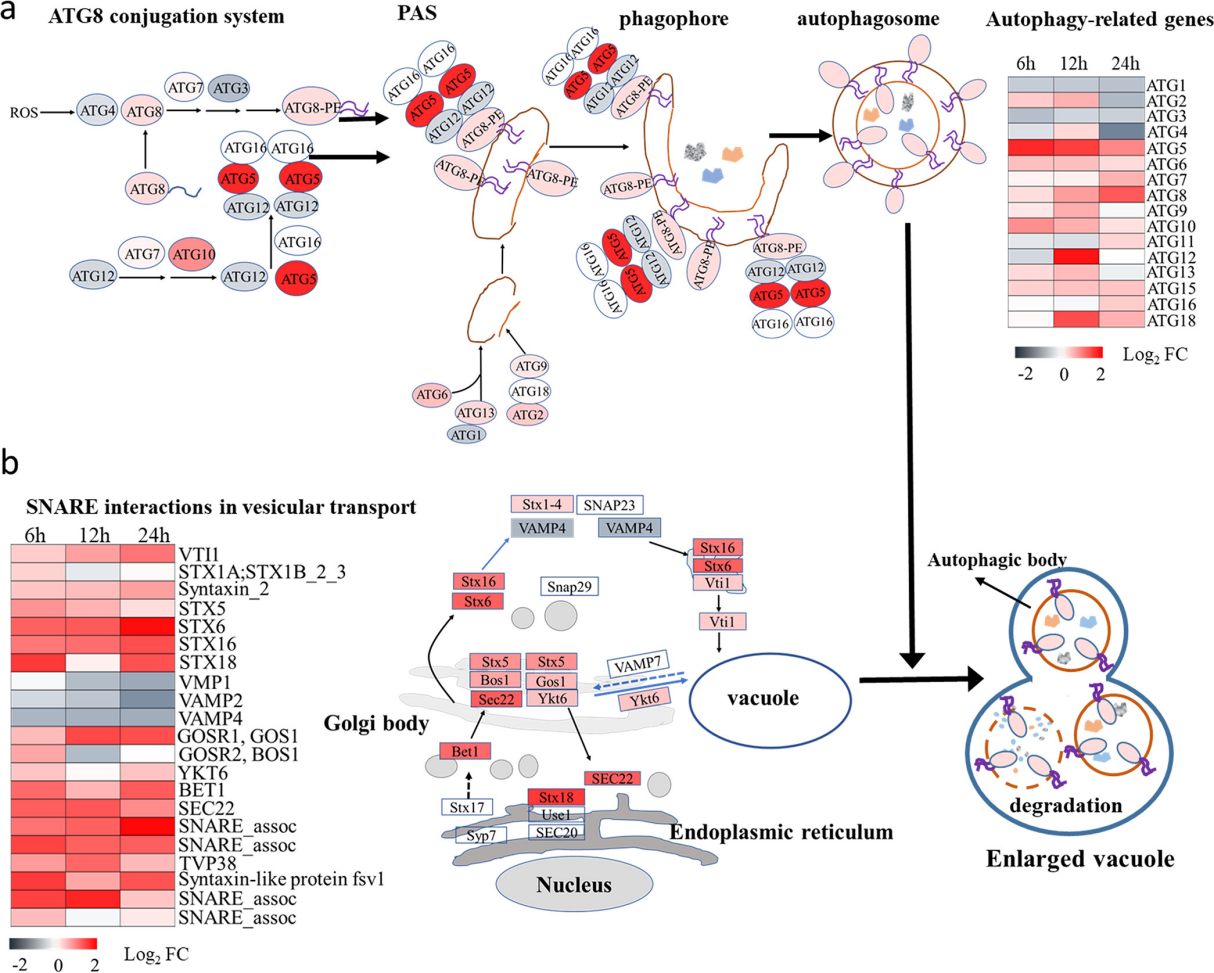
Differentially expressed autophagy-related genes in *P. globosa* treated with the 5% YX04 supernatant. (a) Differentially expressed genes involved in autophagosome formation. (b) Differential expression of genes related to vesicle transport and autophagosome-vacuole fusion. PAS, phagophore assembly site; SNARE, soluble N-ethylmaleimide-sensitive factor attachment protein receptor.

To verify our speculation, an antibody targeting autophagy-related protein 8 (ATG8) was used to detect autophagic flux by immunofluorescence and Western blotting. As shown in Fig. 9a, the YX04 supernatant induced autophagic flux in *P. globosa* after treatment for 0.5 h. In control cells, small and faint fluorescent spots were scattered, while these fluorescent spots were larger and brighter in YX04-treated algal cells, especially after 9 h, 12 h, and 24 h of treatment. The fluorescence values of the YX04-treated group were 3 to 4.3 times higher than those of the control group (Fig. 9b). Western blotting showed that the protein expression of ATG8 in the treatment groups was markedly increased over time, while its expression in the control groups remained low. In particular, the ratio of ATG8 to ATG8-PE was markedly increased after treatment for 9 h (Fig. 9c and d), indicating the occurrence of autophagic flux. Atg8 is a ubiquitin-like protein that is essential for the synthesis of the double-layer membrane that constitutes the autophagosome vesicle and is responsible for delivering the cargo from the cytoplasm to the vacuole lumen. Transcriptome analysis also indicated that 16 ATGs (ATG1 to ATG13, ATG15, ATG16, and ATG18) in the YX04-treated group were significantly upregulated compared to those in the control group (Fig. 10a). These autophagy-related proteins are involved in the initiation of the ATG8 conjugation system and the formation of phagophore assembly sites (PASs), where autophagosomes form, in the very early stage of autophagy, thereby playing a vital role in autophagy induction. Once autophagy was induced by the YX04 supernatant, transient membrane compartments, called phagophores, formed to envelop the cellular contents. The phagophore expanded and formed an autophagosome. Autophagosomes trafficked to the vacuole, where the outer membrane of the autophagosome fused with the vacuolar membrane, releasing the inner membrane-bound compartment (termed the autophagic body) into the vacuolar lumen. The autophagic body and its contents were broken down (Fig. 10). Previous studies on plants have confirmed that the ER is the primary phagophore membrane source (42, 43). Recent reports demonstrated that ER and Golgi biogenesis as well as the biogenesis and fusion of autophagosomes with the vacuole requires membrane-bound soluble N-ethylmaleimide-sensitive factor attachment protein receptor (SNARE) (44–46). Our transcriptome analysis also indicated the upregulated expression of SNARE proteins, which included syntaxins (STXs), YKT6, SEC22, BET1, BOS1, and GOS1, and several SNARE-associated proteins (Fig. 10b). Therefore, the immunofluorescence assay and the Western blotting assay of autophagic flux and transcriptome analysis indicated that the YX04 supernatant triggered autophagy in *P. globosa*, which ultimately caused algal cell death.

## DISCUSSION

### Algicidal effects of the YX04 supernatant on *P. globosa*.

During the bloom and decline of red tides, the populations and quantities of marine microbes (bacteria, viruses, etc.) changed significantly, especially in the extinction period. The bacterial abundances and dominant flora changed significantly. Some clades associated with phytoplankton were detected only in algal blooms. Therefore, research on the relationship between bacteria and algae in regard to algal bloom control has become a hot spot, and algicidal bacteria have attracted attention for their ability to control HABs (47). Studies have revealed two modes of algicidal activity, direct and indirect, with the direct mode occurring only when bacterial cells directly contact algal cells (9). Bacterial strains kill algae by secreting extracellular metabolites, and the purified compounds can play effective roles in algal cells, thus constituting the indirect mode (18). In this study, both the bacterial culture and the supernatant of *Microbulbifer* sp. YX04 had efficient algicidal activity, while the bacterial cells had no algicidal activity; therefore, YX04 has indirect algicidal activity. Strains in the *Microbulbifer* genus can degrade complex polysaccharides and synthesize eumelanin (48–51), while the algicidal activities of the *Microbulbifer* genus are reported less often. The algicidal substances produced by strain YX04 accumulated during the cultivation process, and its algicidal activity peaked during the late-stationary stage. In addition to *P. globosa*, YX04 could effectively kill other red tide-causing species, including T. pseudonana, T. weissflogii, S. costatum, H. akashiwo, and P. donghaiense, thus indicating its broad algae-killing spectrum and its potential for use to control HABs caused by these algal species. Interestingly, YX04 had no algicidal effect on any of the tested algal species in Chlorophyta. According to previous reports, we concluded that it is probably related to the cell wall structure of these species. Cell walls are the outer boundary of algal cells that interact directly with the external environment. The cell wall structure determines the extent of contact with the surrounding environment and plays a critical ecological role in protection against biotic and abiotic stress. Green microalgae have three different types of outer cell wall structures with regard to low, medium, and high chemical resistance (52). Medium-resistant (MR) and high-resistant (HR) cell wall structures were found in several species of the genera *Acutodesmus*, *Botryococcus*, *Chlorella*, *Desmodesmus*, *Nannochloropsis*, and *Scenedesmus* (53–55). An important chemical structure of cell wall resistance is the biopolymer algaenan, which is highly resistant to alkali/acid hydrolysis and aqueous/organic solubilization (52, 54). Algaenan is part of the trilaminar structure/sheath, where the cell wall usually consists of 10- to 20-nm-thick sandwich-like layers with two outside layers of high and one inside layer of low electron density (52). Algae with thick algaenan-containing cell walls survive the gut passage by daphnid feeders due to the resistance of algaenan to gut enzymes (56). The genera *Botryococcus* and *Nannochloropsis*, with promising high intracellular oil content but algaenan-containing cell walls, have cell walls that are especially difficult to disrupt and even hinder acetolysis (54). Therefore, much more energy is required for the biotechnological extraction of intracellular compounds from these HR species. Due to different chemical properties of a highly resistant cell wall structure, the cell is highly protected not only against enzymatic and chemical attack by viruses, bacteria, fungi, or zooplankton but also against interference interaction with phytoplankton species (55, 57, 58). Microcystis aeruginosa could impair the growth of Oocystis marssonii and Chloroidium saccharophilum belonging to the low-resistance (LR) cell wall type, while MR (Chlorella vulgaris) and HR species (Acutodesmus obliquus and Desmodesmus armatus) were not affected in coculture with *M. aeruginosa*. The MR and HR cell walls definitively acted as a structural barrier (52, 53). In our tested algal species in Chlorophyta, *Chlorella vulgaris* and Nannochloropsis gaditana definitively belong to MR and HR species. According to above reports, their algaenan-containing cell walls protect them against YX04 attack.

This study is the first to demonstrate that *Microbulbifer* sp. YX04 effectively killed the unicellular phytoplankton *P. globosa* by extracellular metabolites. In addition, the YX04 supernatant showed an excellent algicidal activity after exposure to various pH values (pH = 4 to 12) and temperatures (−80 to 100°C) for 2 h (Fig. S2), indicating the practical storage advantage of YX04 algicidal substances under temporarily adverse environmental conditions (59). To further address the algicidal mechanism and characteristics of strain YX04 for practical application in HAB control, we isolated and identified algicidal substances from the YX04 supernatant via size fractionation, mass spectral analyses, and nuclear magnetic resonance spectroscopy. According to our recent preliminary result, the high algicidal activity of strain YX04 is a comprehensive effect carried out by at least seven compounds, which are probably related to cyclic dipeptides and diones. The real nature of these compounds needs further elucidation.

### Transcriptome analysis revealed the gene regulated response of *P. globosa* to the YX04 algicidal process.

To explore the algicidal mechanism of the YX04 supernatant, we performed *P. globosa* transcriptome analysis during the algicidal process. While no genomic data are currently available for *P. globosa*, 11,312 unigenes were herein shown to have enriched functional annotations by Gene Ontology analysis, with *Arabidopsis* and Chlamydomonas reinhardtii serving as the references. At level 2, the unigenes were annotated with three functions: BP, CC, and MF (Fig. S2). Among the upregulated genes, most of the top 20 GO terms were associated with biogenesis, repair, replication, biosynthesis, and metabolism. These functions were mostly related to self-repair and survival. This result indicated that algal cells produced a series of stress responses triggered by the YX04 supernatant to prevent self-damage. When algal cells are stimulated by external pressure, related genes are up- or downregulated in response to stress (60). The downregulated expression of genes related to photosynthesis, respiration, antioxidant enzymes, and movement verified the effect of YX04 on algal cell morphology, organelles, and physiological and oxidative stress (Fig. 5 to 7). These functions were closely related to cell self-repair and escape adversity. The long-term continued downregulation of those genes hindered the self-repair function and made the resumption of activity in damaged cells difficult.

### Strain YX04 induces irreversible morphological and structural damage in *P. globosa*.

Compared with those of the control, the flagella of the YX04-treated group were absent, and the shapes and sizes of the cells were significantly different. The dense lamellar structure of thylakoids was destroyed, which affected photosynthesis by decreasing electron transport, photon absorption, and the PS II reaction center in algae (16). Under the stress of strain YX04, algal cells were deformed and ruptured, their internal structure was destroyed (Fig. 7), and tubulin-related genes (TUA and TUB genes) were significantly downregulated (Fig. 7e). Tubulin proteins are major components of ciliary microtubules and the principal protein subunit of microtubules in the cell cytoplasm (61). Under adverse conditions, microtubule-related genes were downregulated, and the cell structure was irreversibly damaged, which seriously affected cell morphology and functions. Two clear flagella were observed in healthy cells of the 2216E group (Fig. 7a), while the flagella of the YX04 group were blurred (Fig. 7b to d). Transcriptome analysis showed that the movement-related genes (most of the IFT family and dynein genes) in the YX04 group were also significantly downregulated (Fig. 7f). IFT family genes have been discovered for many years and play an important role in flagellum assembly (62). The disappearance of the flagella in the YX04 treatment group indicated not only mechanical damage but also impaired flagellum assembly. Since flagella are known as sense organs (62), their disappearance and impaired assembly most likely constitute a response to adverse conditions to protect cells against damage, such as how house lizard tails are broken to promote escape from danger.

### Strain YX04 caused oxidative stress in *P. globosa*.

ROS include single oxygen species (^1^O_2_), superoxide radicals (O_2_^•−^), hydrogen peroxide (H_2_O_2_), and hydroxyl radicals (•OH), which are produced in the mitochondrial electron transport chain, chloroplasts, and peroxisomes (20). ROS can oxidize various biomolecules, including lipids, DNA, and proteins, and cause severe oxidative damage to cell and organelle membranes. The nonenzymatic (carotenoids, glutathione, etc.) and enzymatic (SOD, POD, CAT, etc.) antioxidant defense systems decrease ROS production and protect biomolecules from deleterious ROS effects. When the formation of ROS exceeds the capacity of antioxidant systems, the oxidation of polyunsaturated fatty acids, amino acids, and nucleic acids by the harmful ROS leads to the damage of cellular compartments and consequent cell death (63).

In chloroplasts, the formation of O_2_^•−^ occurs during the light reaction of photosynthesis, and the formation of H_2_O_2_ is caused by the water splitting manganese complex in PS II (64). Photosynthesis is a multistep process involving successive redox reactions, and photosynthetic electron transport depends on the structure of the thylakoid membrane. Molecular oxygen is reduced by the highly reducing cofactors on the acceptor side of both PS II and PS I. When water undergoes photolysis by PS II to activate electron transport, PS II, proton enthalpy, Cytb6/f, plastocyanin, PS I, and NADP^+^ eventually produce NADPH and simultaneously transfer protons into the thylakoid, thereby promoting ATP synthase to generate ATP (65). In algal cells under adverse conditions, the electrons from H_2_O are transferred to Fd via PS II and PS I and are then transferred to O_2_ to form superoxides, which constitute one source of ROS production in the thylakoid. Consequently, less NADP^+^ is reduced, which slows down the rate of CO_2_ fixation (66). Two photosynthetic parameters, Fv/Fm and rETR, are frequently used to characterize the maximum quantum efficiency of PS II photochemistry (67) and the proportion of open oxidized reaction centers in PS II (68). Exposure to the YX04 supernatant significantly decreased the Fv/Fm and rETR of *P. globosa* compared with those of the control group (Fig. 5), which indicated that the photosynthetic efficiency and capacity of algal cells were significantly inhibited. The expression of genes in the photosynthetic system was significantly decreased in the YX04-treated group at the transcriptome level (Fig. 5f), further affecting the biosynthesis of photosynthetic pigments and the regeneration of photosynthetic electron transport chain complexes, which eventually resulted in the complete disruption of photosynthesis. The decreasing Fv/Fm and rETR values and increasing ROS levels (Fig. 3a) led to the speculation that the electron transport chain was blocked, thereby leading to excessive ROS production. The accumulation of ROS caused oxidative stress in the thylakoid environment and changed the characteristics of the thylakoid membrane; electrons did not pass to F-type ATPase and NADP^+^, which ultimately affected carbon fixation. Therefore, the process caused irreversible damage to algal cells.

Mitochondria are an important source of ROS. The superoxide anion radical (O_2_^•−^) is known to be formed by complex I and complex III (40). Complex II was not traditionally regarded as a source of ROS. However, it is associated with increased ROS production when the catalytic activity of complex II switches from succinate dehydrogenase to fumarate reductase at reduced oxygen tension (69). According to transcriptome analysis (Fig. 6), the downregulated expression of genes related to respiratory electron transport chains (complexes I to IV) inhibited the regeneration and repair of the mitochondrial respiratory chain, which caused decreased reduction of electron transport through the mitochondrial respiratory chain and triggered ROS overproduction. The magnitude of the transmembrane electric potential regulates ROS generation by the respiratory chain (70). The markedly downregulated expression of ATPase genes (Fig. 6) strengthened the magnitude of the transmembrane electric potential, which increased the oxidative stress in mitochondria. The marked continuous downregulation of the mitochondrial SOD2 (SOD-Mn) gene indicated that O_2_^•−^ was not scavenged in time and resulted in obvious accumulation of mitochondrial superoxide level. Mitochondria, as both generators and targets of ROS, accumulate some of the damage that can initiate a malignant circle of further ROS formation.

ROS activate antioxidant enzymes, including SOD, CAT, and POD. Primarily, O_2_^•−^ is converted to H_2_O_2_ by SOD. Subsequently, CAT and POD remove H_2_O_2_ to protect the cells from oxidative damage (71). The treatment of algal cells with bacteria or algicidal compounds resulted in excessive ROS production, thereby causing lipid peroxidation, photosynthetic oxidation, and, ultimately, cell death (22). The levels of ROS and H_2_O_2_ were significantly increased (Fig. 3a and b), indicating high levels of oxidative stress in *P. globosa* caused by the YX04 supernatant. In addition to the damage to the photosynthetic system, many genes related to respiratory electron transport chains were downregulated in algal cells after treatment with the YX04 supernatant (Fig. 6), indicating the dysfunction of respiratory systems. The dysfunction of electron transport in both chloroplasts and mitochondria caused ROS overproduction and the formation of oxidative stress. KEGG enrichment analysis also indicated the significantly upregulated expression of peroxisome-related genes (Fig. 4c), suggesting ROS overproduction. Oxidative damage has also been associated with a decline in antioxidant defense efficiency, which together with increased ROS production significantly contributes to the manifestation of an oxidative stress state. To relieve oxidative damage from ROS, the antioxidant enzyme SOD was activated (Fig. 3c) and SOD1 and SOD-Cu gene expression was upregulated (Fig. 6). The activities of SOD at 9, 12, and 24 h were significantly higher than the initial levels. Despite the increasing activities of SOD, the overproduction of O_2_^•−^ was not completely cleared in time (Fig. 3). The product of SOD scavenging O_2_^•−^ is H_2_O_2_, and the SOD activity in algal cells treated with the YX04 supernatant peaked at 9 h, which was earlier than the peaks of H_2_O_2_ and MDA contents. CAT and POD remove H_2_O_2_ to protect the cells from oxidative damage. The downregulated expression of POD and CAT genes (Fig. 6b) decreased the activities of POD and CAT, suggesting that H_2_O_2_ reduced by SOD was not completely removed and resulted in the accumulation of H_2_O_2_. H_2_O_2_ accumulation further increased the oxidative stress formed by O_2_^•−^ overproduction in chloroplasts and mitochondria. The high MDA content in *P. globosa* confirmed that excessive ROS (O_2_^•−^ and H_2_O_2_) triggered lipid peroxidation (Fig. 3d). Transcriptome analysis indicated the significantly upregulated expression of genes related to glycerophospholipid and glycerolipid metabolism (Fig. 4f), suggesting the occurrence of lipid peroxidation. Lipid peroxidation disrupts the functions of many fundamental organelles and structures (18). Zhang et al. (21) reported that lipid peroxidation triggered by Sulfitobacter porphyrae ZFX-1 markedly increased the proportions of saturated fatty acids (SFAs) and decreased the degree of total lipid unsaturation in *P. donghaiense*. These phenomena led to large amounts of SFAs forming cell membranes and to cell membrane solidification, which destroyed the function of the membrane system, including the thylakoid and plasma membranes. Although the GSH content was not detected herein, the GSH metabolism pathway was significantly upregulated (Fig. 4c), suggesting that the nonenzymatic antioxidant GSH also probably plays important roles in scavenging ROS.

### The mode of death and mechanism of *P. globosa* triggered by strain YX04.

Autophagy is a cellular degradative pathway in which numerous cargoes, including protein aggregates, damaged organelles, and invading pathogens, are targeted to the vacuole or lysosome for degradation and recycling. Basal autophagy is important in balancing anabolic and catabolic pathways and is therefore required to maintain general cell homeostasis. In addition, autophagy is an effective defense mechanism against pathogens and recycling machinery for macromolecules that helps the cell cope with nutrient scarcity. Autophagy is induced by nutrient limitation, and the degradation of ribosomal proteins is used for the accumulation of triacylglycerols in Chlamydomonas reinhardtii (72). Along with its survival role, autophagy can lead to programmed cell death upon severe stress. Under severely adverse conditions, autophagy is one mode of cell death that is regulated by autophagy-related proteins and leads to autophagosome formation (59). A number of double-membrane vesicles in the YX04-treated group were observed by TEM (Fig. 8); however, the same vesicles were not found in the control group (Fig. 7a and e). Double-membrane vesicles were produced in the cytoplasm and degraded in the vacuole (Fig. 8h, red arrow), which closely resembled the production and degradation of autophagosomes in plants or other species (73, 74). A cup-shaped double-membrane phagophore was formed around unwanted proteins and damaged organelles. The membrane elongated and closed to form a double-membrane vesicle, the autophagosome. Autophagosomes were transported into vacuoles, which were degraded by resident hydrolase and recycled. These double-membrane vesicles were subsequently identified as acidic autophagosomes by an immunofluorescence assay (Fig. S3 and Fig. 9). Analysis of ATG8 localization by immunofluorescence revealed that the ATG8 protein appeared as weak fluorescent dots in the control cells. However, the ATG8 fluorescent dots appeared as ellipses in the treated cells, and the intensity was enhanced as the treatment time was extended (Fig. 9a). The number and sizes of the immunofluorescent spots significantly increased in response to treatment with the YX04 supernatant, reflecting the initiation of autophagy and the formation of autophagosomes. Therefore, we speculated that the *P. globosa* cells in the YX04 treatment group underwent autophagic cell death. The occurrence of autophagic flux (Fig. 9) and the upregulated ATG expression (Fig. 10) verified our speculation.

To date, more than 30 ATG genes have been identified in various macroautophagy subtypes, which are conserved from yeasts to plants and mammals (75) and in the green alga Chlamydomonas reinhardtii (30). ATG genes encode proteins as the “core autophagic machinery” and participate in formation of the autophagosome (30). Among ATG proteins, the ubiquitin-like protein ATG8 plays a pivotal role in the processes of autophagosome formation, target recognition, and vacuole tethering. ATG8 functions by covalently binding to the autophagosome lipid membrane phosphatidylethanolamine (PE). The lipidation of ATG8 to PE is catalyzed sequentially by the ATG8 conjugation system, which includes the cysteine-protease ATG4, the ATG12–ATG5–ATG16 complex, and enzymes (Fig. 10a). Finally, ATG8-PE is released from the outer autophagosome membrane via the delipidating function of ATG4, and the portion wrapped by the inner membrane as the autophagic body is degraded in the vacuole (Fig. 10b). The two isoforms of the ATG8 protein, ATG8 and ATG8-PE, have been widely used to monitor the process of autophagosome formation in different organisms, including algae. In *Chlamydomonas*, a specific ATG8 antibody can be used to distinguish the two types of ATG8 by Western blotting because ATG8-PE migrates faster than the unmodified protein (30). Fortunately, we detected distinct bands of ATG8 (Fig. 9c) in *P. globosa* using the purchased anti-ATG8 antibodies from Chlamydomonas reinhardtii. Compared with that in the control group, the ratio of the ATG8-PE protein to the ATG8 protein in the treatment group was significantly increased, indicating obvious autophagic flux. Even after 24 h of treatment, the expression level of ATG8 remained high, while most of the algal cells had died. Thus, we believe that the *P. globosa* cell death triggered by strain YX04 was caused by excessive autophagy. Yu et al. reported that the algicidal compounds secreted by *Streptomyces* sp. U3 possibly caused autophagy in Heterosigma akashiwo (76). They observed only double-membrane vesicles in algal cells and did not detect autophagic flux. Therefore, they did not confirm that algal cell death was caused by autophagy. Autophagy is a normal physical process and has been reported to have both prosurvival and prodeath roles in the regulation of hypersensitive responses and plant immune responses to biotic stresses. Only continued excessive autophagy can cause cell death. We detected large-scale autophagic flux in *P. globosa*, and the autophagy process continued for a long time. Therefore, we suggest that the cell death of *P. globosa* triggered by *Microbulbifer* sp. YX04 is due to excessive autophagy.

### Conclusion.

**FIG 11:**
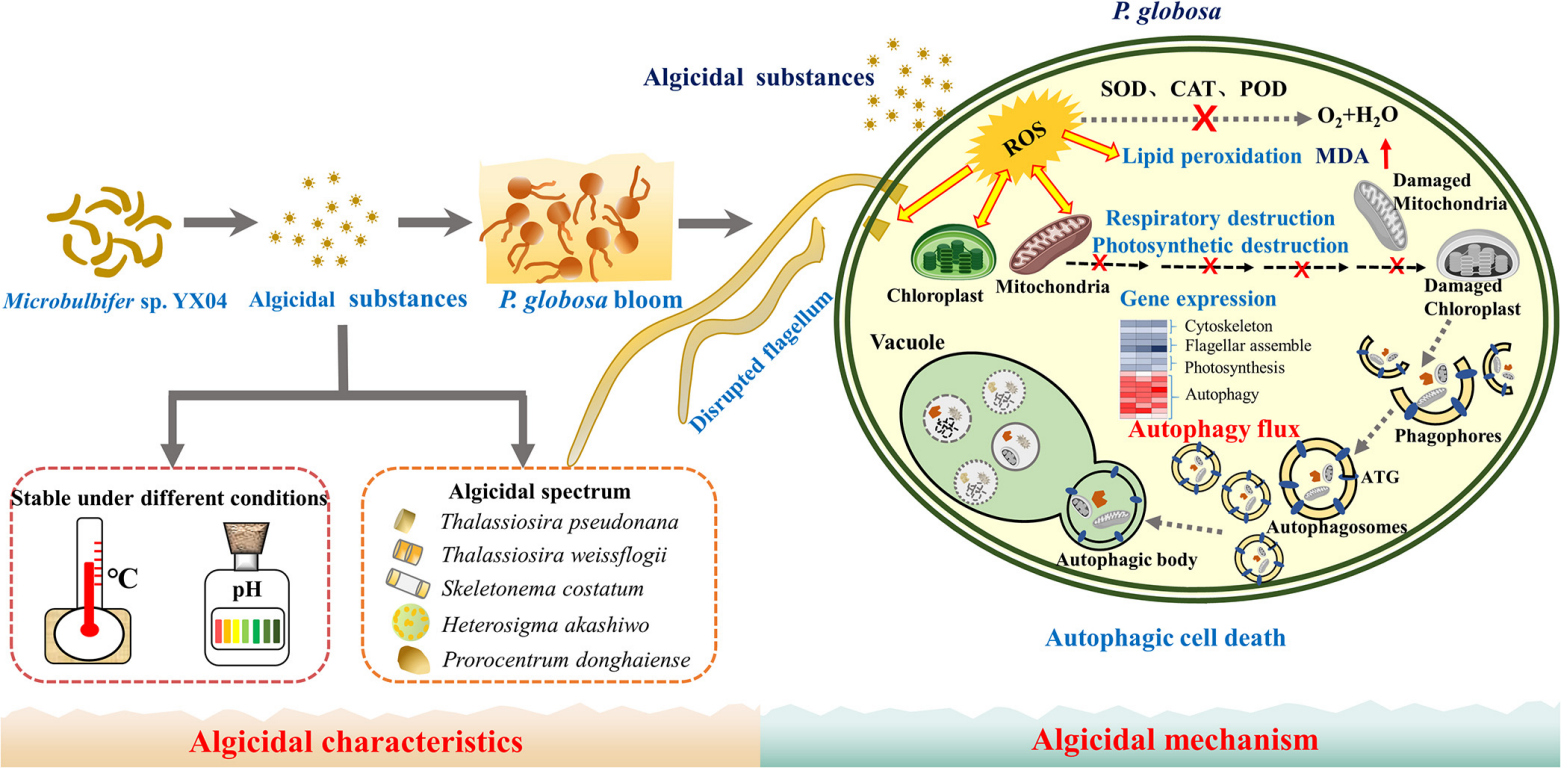
A schematic diagram depicting the algicidal mechanism of strain YX04 against *P. globosa*. ATG, autophagy-related proteins.

*Microbulbifer* sp. YX04 obviously exerted an indirect algicidal effect on *P. globosa* by producing and secreting algicidal substances. The YX04 supernatant caused oxidative stress, which induced lipid peroxidation, damaged the membrane system, and markedly altered global gene expression in *P. globosa*. Membrane system damage and downregulated expression of genes related to photosynthesis and respiration inhibited the primary photochemical reaction and blocked photosynthetic and respiratory electron transport, which caused ROS overproduction. Although the increase of SOD activity and upregulated expression of SOD1 and SOD-Cu scavenged a portion of the ROS, the downregulated expression of SOD2, CAT, and POD resulted in the accumulation of O_2_^•−^ and H_2_O_2_, which strengthened the oxidative stress status. The downregulated expression of cytoskeleton- and flagellum-related genes resulted in morphological changes and motility loss in algal cells. Cellular oxidative stress and structural organelle destruction induced the upregulated expression of genes related to autophagosome biogenesis (ATGs) and vacuole fusion in *P. globosa*, leading to the formation of autophagosomes, which engulfed and trafficked the damaged cellular structures to vacuoles for digestion (Fig. 11). The continuous autophagic process ultimately led to algal cell death. Therefore, strain YX04 induces oxidative damage and autophagic death in *P. globosa*. In addition to *P. globosa* of Chrysophyta., strain YX04 exhibited high algicidal activity against 5 different algal species in Bacillariophyceae, Xanthophyceae, and Pyrrophyta and thus could be useful for controlling HABs caused by these algal species. The broad algicidal spectrum, acid-base tolerance, and temperature stability of algicidal substances make strain YX 04 promising for the microbial control of HABs in the future.

## MATERIALS AND METHODS

### Algal strain and culture.

*P. globosa* CCMA-191 was kindly provided by the National Key Laboratory of Marine Environmental Science of Xiamen University. All cultures were maintained in f/2 medium at 20 ± 1°C under a 12-h light/12-h dark cycle with a light intensity of 50 μmol photons m^−2^ s^−1^. Strain YX04, isolated at the Yunxiao Mangrove National Nature Reserve in Fujian Province, was incubated in Zobell 22l6E broth at 30°C.

### Identification of the algalytic activity of the bacterial strain YX04.

Strain YX04 was characterized by the 16S rRNA gene according to our previous report (21). The related sequences were obtained from the EzTaxon-e and GenBank databases for sequence alignment. Phylogenetic analysis was performed using MEGA 5.1 software. Evolutionary distance analyses were performed using the neighbor-joining method (77). Bootstrap values were evaluated based on 1,000 replications. The cell morphology was observed using transmission electron microscopy (JEM2100HC, JEOL Co., Japan).

### Assay for determining the algicidal activity of strain YX04.

Bacterial cultures (1-mL aliquots) were added to 20 mL of *P. globosa* cultures in the axenic logarithmic growth phase. Algal culture growth was monitored by measuring chlorophyll autofluorescence at an excitation wavelength of 440 nm and an emission wavelength of 680 nm. The algicidal activity was calculated using the following equation.

$$\text{algicidal activity}\left(\%\right)=(\text{1}-F_{\text{t}}/F_{0})\times \text{1}00$$

*F*_t_ and *F*_0_ represent the fluorescence values of the algal cultures at different time points and at the beginning of the treatment period, respectively. Algal culture supplemented with 5% 2216E broth served as the control. All treatments were repeated three times.

### Assay for determining the algicidal mode of strain YX04.

Strain YX04 was inoculated in 100 mL of 2216E broth and grown to stationary phase at 30°C while shaking at 150 rpm for 48 h. The cell-free supernatant of the YX04 culture was collected by centrifugation at 12,000 × *g* for 10 min and then filtered through a 0.22-μm Millipore membrane. The cell pellets were washed twice with 2216E broth and then resuspended in the same volume of sterile f/2 medium. The different fractions of the YX04 cultures were added to algal cultures at a volumetric fraction of 5% to measure their algicidal activity. The same volume of 2216E broth was added to algal cultures as a control. The algicidal activity was calculated according to the formula above.

### Transmission and scanning electron microscopy observation.

The algal cells were collected by centrifugation at 3,000 rpm for 5 min and washed twice with a 0.01 M phosphate-buffered saline (PBS) solution (NaCl 8 g/L, KCl 0.2 g/L, Na_2_HPO_4_ 1.44 g/L, KH_2_PO_4_ 0.24 g/L [pH 7.4]). Then, the cells were fixed with 2.5% glutaraldehyde in 0.01 M PBS for 12 h at 4°C. For SEM observation, the fixed cells were attached to a cover slip that was precoated with 0.5% formvar as the adhesive, dehydrated in a graded ethanol series (30, 50, 70, 80, 95, and 100%), and then dried at the critical point. The dry cells were sputter-coated with gold on a copper mesh and imaged via SEM (JSM-6390LV, JEOL Co., Japan). For TEM observation, the algal cells were gently embedded in 2% agar blocks, which were then fixed with glutaraldehyde solution (2.5%) overnight. After washing three times with 0.01 M PBS, the cells were postfixed in 1% OsO_4_ (vol/vol in 0.1 M PBS) for 2 h and then dehydrated in a graded ethanol series (30, 50, 70, 90, and 100%). The samples were embedded in LR white resin. The resin block was cut into 70-nm-thick ultrathin sections with a Leica Mltracut R microtome (Leica, Germany), double-stained with 2% uranyl acetate-lead citrate on a copper mesh, and then observed using TEM.

### Photosynthetic activity measurement.

Algae cultures treated with 5% YX04 supernatant for 6, 12, 24, and 48 h were used for the measurements of the maximum photochemical quantum yield (Fv/Fm) of photosystem II (PS II) and the relative electron transfer rate (rETR) using a pulse-amplitude modulation fluorometer (Multi-Color-PAM, WALZ, Germany). The Fv/Fm of the algal cells was measured after incubation in the dark for 15 min.

### Detection of ROS levels and malondialdehyde content.

Algal cultures were treated with 5% YX04 supernatant, and samples were collected after 0, 3, 6, 9, 12, and 24 h of treatment; algal cultures mixed with 5% 2216E served as the control. The ROS level was detected using the 2′,7′-dichloro-fluorescein diacetate (DCFH-DA) probe, and DCFH-DA fluorescence was assessed according to Zhuang’s method (20). The level of H_2_O_2_ in ultrasonically lysed cells was detected by diagnostic reagent kits. The MDA content was measured as the peroxidation index with an MDA assay kit according to the manufacturer’s instructions. All test kits were supplied by Beyotime Biotechnology (Shanghai, China). The protein content was measured using a total protein quantitative assay kit (Nanjing Jiancheng Bioengineering Institute, China) and bovine serum albumin as the standard.

### Transcriptome analysis.

Exponential-phase algal cultures were treated with 5% YX04 supernatant or 5% 2216E broth for 6, 12, and 24 h. Total RNA was extracted from the collected algal cells by TRIzol (Invitrogen) and detected by 1% agarose gel electrophoresis. RNA samples of sufficient quality were used to prepare a cDNA library, which was sequenced on the Illumina HiSeq 4000 platform (Illumina Inc., San Diego, USA) by Majorbio Technology Inc. (Shanghai, China). Raw sequencing reads were screened with SeqPrep (https://github.com/jstjohn/SeqPrep) and SICKLE software (https://github.com/najoshi/sickle) to remove low-quality or default reads. All of the obtained reads were assembled into contigs and singletons. Open reading frame (ORF) prediction was carried out using the novel method TRINITY (https://sourceforge.net/projects/trinityrnaseq/). Gene expression (fragments per kilobase per million [FPKM]) and differential expression levels between the control group and the YX04-treated group were analyzed using EDGER software (http://www.bioconductor.org/packages/2.12/bioc/html/edgeR.html) and DESeq2 (with biological duplication). Differences were determined based on the read count data of the compared genes and the negative binomial distribution model. Genes that met the default screening criteria of a *P*-adjusted value of <0.05 and a |log_2_FC| of ≥1 were regarded as differentially expressed genes (DEGs). All unigenes were aligned to the NCBI nonredundant (nr) protein sequence database, STRING, and Swissprot database using BLASTx search. Functional annotation and classification of all of the unigenes were conducted using the Gene Ontology database (GO; http://www.geneontology.org/) and the Kyoto Encyclopedia of Genes and Genomes databases (KEGG; https://www.kegg.jp/). The KEGG Automatic Annotation Server (KAAS; https://www.genome.jp/tools/kaas/) was used to assign orthologues and map pathways represented in the *P. globosa* transcriptome. The GO terms and KEGG pathways with a *P* value of <0.05 were considered to be significantly enriched GO terms and KEGG pathways, respectively. All of the up- or downregulated unigenes described in the present study are illustrated by the first comparison component. GOSeq and KOBAS software were used to estimate the statistical enrichment of DEGs in GO terms and KEGG pathways, respectively Transcriptome analysis was performed using the free online Majorbio I-Sanger Cloud Platform (https://cloud.majorbio.com/).

### Immunofluorescence detection.

Algal cells were collected by centrifugation at 3,000 rpm for 5 min, washed twice with 0.01 M PBS (pH 7.4), and then resuspended in PBS. The collected algal cells were placed on a dried coverslip coated with 1% polyethyleneimine and allowed to adhere for 2 min. Excess medium (not completely dry) was aspirated, and the coverslip was immersed in methanol precooled to −20°C. The coverslips were placed in methanol in a refrigerator for 10 min and quickly transferred to −20°C ethanol. After 5 min, the coverslips were removed from the ethanol and air dried before being placed on coverslips in blocking buffer (5% [wt/vol] bovine serum albumin [BSA], 1% [vol/vol] fish skin gelatin [Sigma-Aldrich Co., USA], 10% [vol/vol] goat serum [Sigma]). PBST (PBS plus 0.05% Tween 20) was added to the coverslips for at least 30 min, followed by incubation with a primary anti-ATG8 antibody (no. AS142769, Agrisera, Sweden; diluted in blocking buffer 1:1,000) overnight at 4°C. The coverslips were washed 4 times with PBST for 5 min each and incubated with an Alexa Fluor 488-labeled goat anti-rabbit antibody (no. A11008, Thermo Fisher; 1:2,000 dilution) in the dark for 2 h at room temperature. The cells were washed 3 times with PBST for 5 min each, immersed in 100% ethanol for 1 min, and then air dried. Mounting medium warmed to room temperature was placed on a microscope slide (20 μL), followed by the gentle placement of a cover slip (cell side down) fixation with nail polish. Fluorescence signals were observed and photographed on a laser scanning confocal microscope (Zeiss LSM780, Carl Zeiss, Germany). For comparative analysis, the same acquisition time was used for the Alexa Fluor 488 signals. The relative immunofluorescence intensity of ATG8 was analyzed using ImageJ software (78) to calculate the relative brightness of each sample cell. The same parameters were used for the background to ensure that the obtained values reflected the fluorescence intensity of ATG8 in each cell. The mean values were determined from 30 cells per sample, and the error bars indicate the standard errors of 30 cells.

### Protein preparation and Western blotting.

*P. globosa* cells were collected by centrifugation at 3,000 rpm for 5 min, washed twice with 0.01 M PBS (pH 7.4), and resuspended in 500 μL of TRIzol (TaKaRa, Japan). The cells were sonicated (100 W, ultrasonication time of 5 s and rest time of 5 s for 90 cycles) using the Ultrasonic Cell Disruption System (Ningbo Scientiz Biotechnological Co., Ltd., China). Proteins were precipitated from the organic phase with isopropanol (80109218, Sinopharm Chemical Reagent Co., Ltd., China) and collected by centrifugation at 10,000 × *g* for 8 min at 4°C. Then, the proteins were resolubilized in 8 M urea and quantitated by the Coomassie blue dye binding method. For Western blotting, protein extracts (30 μg) were separated by 15% SDS-PAGE and then transferred to a polyvinylidene fluoride membrane (Millipore Co., USA) previously activated in methanol (Sinopharm Chemical Reagent Co., Ltd., China). The membranes were incubated for 30 min in blocking buffer comprising 5% (wt/vol) nonfat dry milk in Tris-buffered saline (TBS) containing 0.1% Tween 20 (vol/vol). The primary anti-ATG8 (no. AS142769, Agrisera, Sweden) and anti-AtpB (AS05085, Agrisera, Sweden) antibodies were diluted at ratios of 1:2,000 and 1:2,500, respectively, while the secondary goat anti-rabbit antibody (no. A6154/SLBV9141, Sigma, Germany) was diluted 1:5,000. An immunoblotting detection system (C280, Azure Biosystems, USA) was used to detect horseradish peroxidase-conjugated secondary antibodies followed by exposure to ECL reagents (E1050, LABLEAD, China).

### Statistical analyses.

Significant differences among groups in this study were determined by one-way analysis of variance (ANOVA; SPSS 19.0 for Windows), and a *P* value of <0.05 was considered to indicate significance.

### Data availability.

RNA sequencing data were deposited in the Sequence Read Archive (SRA) database (https://www.ncbi.nlm.nih.gov/sra) at NCBI with BioProject accession number PRJNA777965, SRA project accession numbers SRR 16796923 to 16796940, and BioSample accession numbers SAMN22896686 to 22896721.
