# Supplementary material for: A Novel Algicidal Bacterium, Microbulbifer sp. YX04, Triggered Oxidative Damage and Autophagic Cell Death in Phaeocystis globosa, Which Causes Harmful Algal Blooms
Source: Microbiol Spectr. 2022 Jan 12;10(1):e00934-21. doi: 10.1128/spectrum.00934-21 (PMC8754136; doi:10.1128/spectrum.00934-21)
Supplement: SUPPLEMENTAL FILE 1 — Supplemental material. Download SPECTRUM00934-21_Supp_1_seq1.pdf, PDF file, 0.6 MB [file spectrum00934-21_supp_1_seq1.pdf]

# Supplementary materials

## Materials and methods

### Algicidal activity assay of the YX04 supernatant after treatment with different temperatures and pH values

Cell-free supernatants were incubated at -20, 0, 4, 25, 40, 50, 60, 80 and 100 °C for 2 h and then allowed to return to room temperature. The treated supernatant was then inoculated into algal cultures at a 5% (v/v) volume to determine the algicidal activity. The pH values of the YX04 supernatant were adjusted to 3, 4, 5, 6, 7, 8, 9, 10, 11 and 12 using 6 M HCl or 6 M NaOH, and maintained for 2 h. The treated supernatant was adjusted to a pH of 8 and subsequently added to the algal cultures to assess its algicidal activity. The initial YX04 supernatant (25 °C, pH = 8) without any treatment served as the reference group. All experiments were repeated in three biological replicates.

### Assay of the algicidal effects of strain YX04 on other algal species

To investigate whether strain YX04 exerted algicidal effects on other microalgal species, the following 11 algal species were assessed: *Phaeodactylum tricornutum, Thalassiosira pseudonana, Thalassiosira weissflogii, Skeletonema costatum, Amphiprora alata, Heterosigma akashiwo, Dunaliella salina, Platymonas subcordiformis, Nannochloropsis gaditana, Prorocentrum donghaiense* and *Alexandrium minutum.* All cultures were maintained in f/2 medium under a 12 h light/12 h dark cycle with a light intensity of 50 μmol photons $m^{-2}$ $s^{-1}$ at 20±1 °C. Algal cells cultured to the exponential phase (approximately $10^6$ cells/mL) were used for experiments. The YX04 supernatant (5%) was added to each of these algal

cultures. Zobell 2216E served as a control and was added to the algal cultures at the same volume. After 72 h of treatment, the algicidal activity was calculated as described above.

**Detection of acidic vesicles in algal cells**

Algal cells were collected by centrifugation at 3,000 rpm for 5 min, washed twice with 0.01 M PBS (pH = 7.4) and incubated with 500 μL of 100 nM LysoTracker ® Green DND-26 (Thermo Fisher Scientific Co., Ltd, USA) for 5 min at room temperature. After washing twice, the stained samples were observed by fluorescence microscopy and flow cytometry.

# Results

**Table S1 Algicidal spectrum of the YX04 supernatant**

| Strains | Algicidal effects |
|---|---|
| **Bacillariophyceae** | |
| *Phaeodactylum tricornutum* | - |
| *Thalassiosira pseudonana* | + |
| *Thalassiosira weissflogii* | + |
| *Skeletonema costatum* | ++ |
| *Amphiprora alata* | - |
| **Xanthophyceae** | |
| *Heterosigma akashiwo* | ++ |
| **Chlorophyta** | |
| *Dunaliella salina* | - |
| *Platymonas subcordiformis* | - |
| *Chlorella vulgaris* | - |
| *Platymonas helgolandica* | - |
| *Nannochloropsis gaditana* | - |
| **Pyrrophyta** | |
| *Prorocentrum donghaiense* | ++ |
| *Alexandrium minutum* | + |
| **Chrysophyta** | |
| *Phaeocystis globosa* | ++ |

+: obvious algicidal effect; ++: strong algicidal effect; -: no algicidal effect.

**Fig. S1**

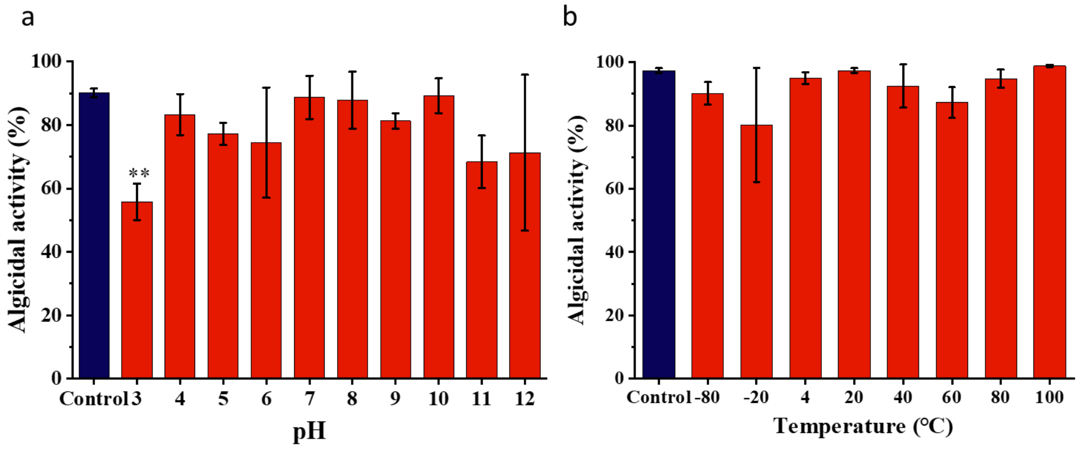


**Fig. S1.** Algicidal stability of the YX04 supernatant at different pH values (a) and temperatures (b). All error bars indicate the SEs of three biological replicates. ** indicates a significant difference at the $p < 0.01$ level compared with the control group.

**Fig. S2**

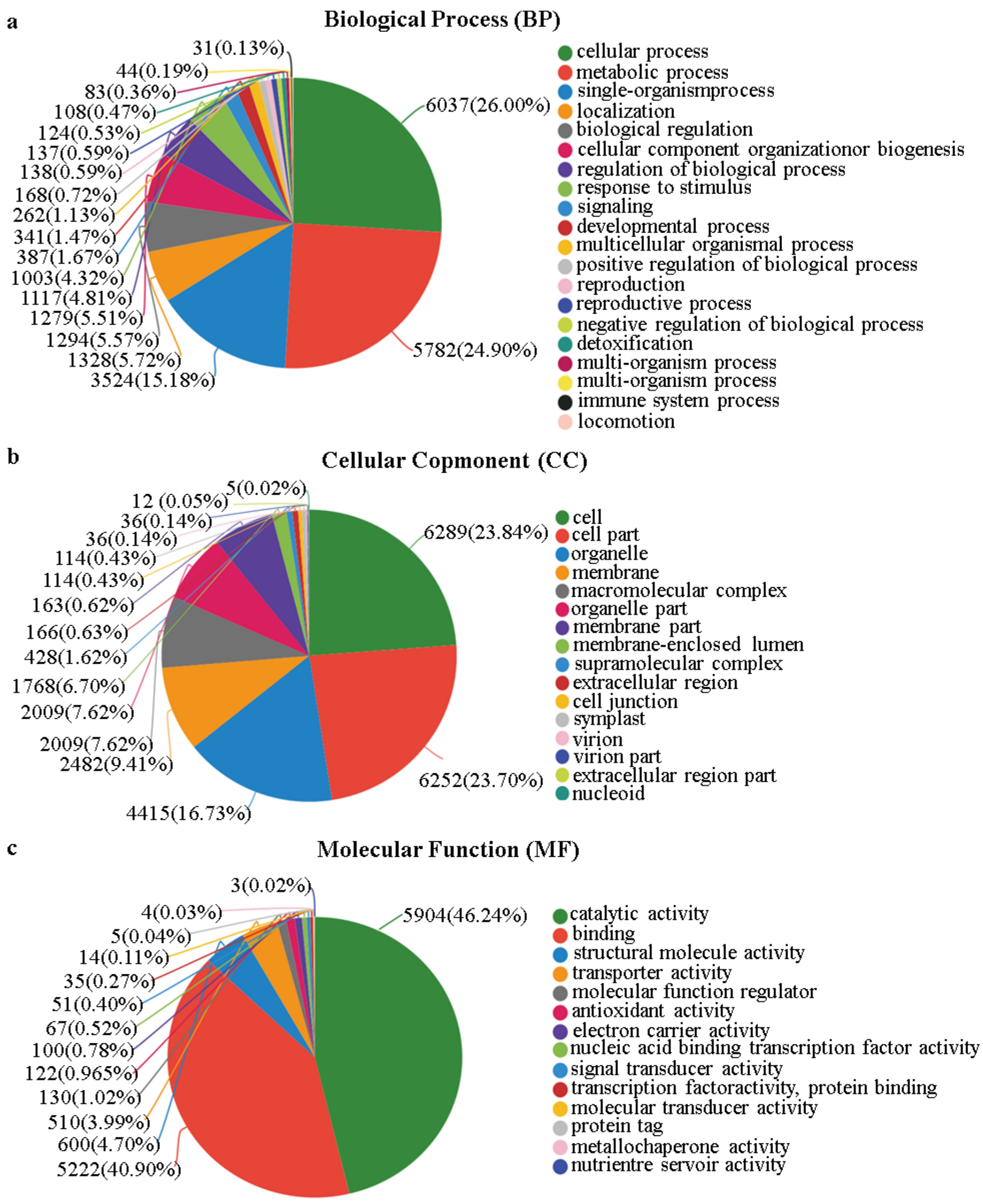


**Fig. S2. GO enrichment of unigenes in *P. globosa* cells.** GO enrichment of 62300 unigenes from all samples. (a) Biological process, (b) cellular component, and (c) molecular function.

**Fig. S3**

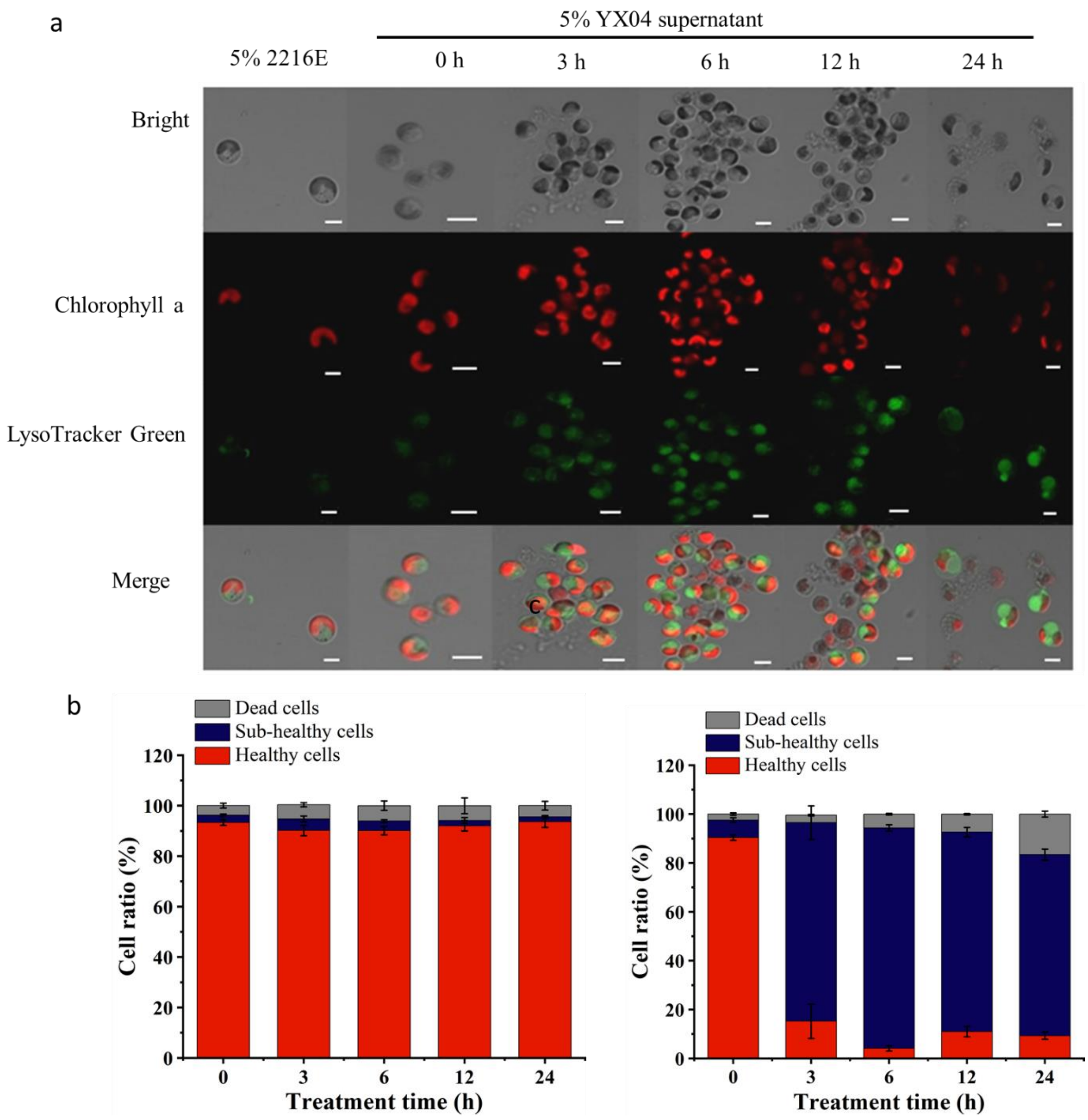


**Fig. S3. Detection of acidic vesicles in *P. globosa* cells treated with the 5% YX04 supernatant.** (a) Observation of cells stained with LysoTrack Green using a fluorescence microscope. (b) Statistical analysis of stained cells in the control group by flow cytometry. (c) Statistical analysis of stained cells in the treatment group by flow cytometry.
